# Wide-ranging barcoding aids discovery of one-third increase of species richness in presumably well-investigated moths

**DOI:** 10.1038/srep02901

**Published:** 2013-10-09

**Authors:** Marko Mutanen, Lauri Kaila, Jukka Tabell

**Affiliations:** 1 Biodiversity Unit, Department of Biology, PO Box 3000, University of Oulu, Finland; 2 Finnish Museum of Natural History, Zoology Unit, PO Box 17, University of Helsinki, Finland; 3 Laaksotie 28, FI-19600 Hartola, Finland

## Abstract

Rapid development of broad regional and international DNA barcode libraries have brought new insights into the species diversity of many areas and groups. Many new species, even within well-investigated species groups, have been discovered based initially on differences in DNA barcodes. We barcoded 437 collection specimens belonging to 40 pre-identified Palearctic species of the *Elachista bifasciella* group of moths (Lepidoptera, Elachistidae). Although the study group has been a subject of several careful morphological taxonomic examinations, an unexpectedly high number of previously undetected putative species is revealed, resulting in a 34% rise in species number in the study area. The validity of putative new species was subsequently supported with diagnostic morphological traits. We show that DNA barcodes provide a powerful method of detecting potential new species even in taxonomic groups and geographic areas that have previously been under considerable morphological taxonomic scrutiny.

Estimates of the number of species on Earth vary from 3 to 100 million, the most recent survey concluding that there are about 8.7 million (±1.3 million SE) species based on a quantitative extrapolation of current taxonomic knowledge^1^. About 1.1 million of the thus far described ~1.9 million eukaryotic species are arthropods, predominantly insects^2^, and the vast majority of arthropod species remains to be described. The huge number of species on Earth and the shortage of taxonomic expertise attending to them have led to the predicament often called the ‘taxonomic crisis’ or ‘taxonomic impediment’^3^,^4^. It has been suggested that, by accelerating species discovery and making species delimitation more straightforward, DNA based tools might be powerful in overcoming the crisis^5^,^6^.

Animal DNA barcode is a short, standardized fragment of the mitochondrial COI gene designed to enable the rapid accurate identification of species and to accelerate species discovery^5^. Similar barcodes, but from different regions of the genome, have been developed both for fungi^7^ and plants^8^. COI has proven efficient in species identification in many species-rich animal groups^9^,^10^, revealing cryptic diversity^11^,^12^, while in others the results have been less promising, with even a large portion of species showing interspecific overlap with closely related species^13^,^14^. DNA barcodes have also been criticized for the shortcomings of the commonly used methodology^15^ and potential problems caused by anomalies of mitochondrial DNA^16^,^17^.

Several factors may have caused considerable biases in estimations of DNA barcode performance. First, in most investigations of species-rich animal groups only a fraction of the species concerned have been sampled. Second, most studies have not examined specimens exhaustively across the distribution ranges, thereby underestimating the level of geography-related intraspecific variability. Third, the sample sizes within populations have often been small, giving an unreliable picture of the distinction between species. While all these factors are likely to produce overoptimistic estimations of the power of barcodes in species identification, other factors may have biased other estimations in the reverse direction. Many papers have examined the power of the barcoding concept in cases which are known *a priori* to be especially difficult (e.g.^18^,^19^). In such cases, barcodes are often compared to the existing morphology-based taxonomy, either explicitly or implicitly considered to represent the “true taxonomy”. A mismatch between barcodes and existing species delineation has been suggested as providing evidence that barcodes do not perform well. Some other studies examining the utility of barcodes have been based largely on data uploaded from GenBank, which is known to include a high degree of misidentifications^20^,^21^. Some of those investigations are among the ones that have presented the lowest identification success and the highest rates of intraspecific variability of barcodes^13^,^19^,^22^.

Tests of the efficacy of barcodes in species identification always reflect the opinions of investigators (or the taxonomists behind the reference taxonomy) and are prone to some subjectivity, at least in the treatment of allopatric populations (cf.^23^), and to a variety of existing species definitions. Therefore, even identical data sets might easily lead to different conclusions. An example of this is the *Astraptes fulgerator* complex of skipper species, in which the same data led different investigators to deviant estimations of the number of species involved^11^,^24^. We recognize that barcodes have their limitations, as differences in the mitochondrial DNA sequences are not the cause, but rather a result, of a speciation event, and the formation of discrete haplotype clusters between the species generally requires time. Thus, the existence of even considerably different haplotypes *per se* is not sufficient evidence for basing taxonomic decisions^25^. Young species are even unlikely to be differentiated by barcoding alone, as it can be suggested that they share more or less the same haplotype pool as the ancestral taxon. Thus, in addition to a discrete barcode, a taxon can only be recognized as a distinct species with the support of other evidence, be that molecular (e.g. nuclear markers), morphological, ecological, or the proven absence of successful interbreeding as shown by the absence of panmictism. Still, there is mounting evidence that generally, at least on a local scale, different, established species usually possess their own cluster of barcode haplotypes^22^,^23^,^24^,^25^,^26^,^27^,^28^,^29^,^30^,^31^.

Wide adoption of molecular techniques in taxonomic research has significantly increased the recognition of cryptic species, i.e., species that have remained previously undetected because of close relatedness and morphological similarity^32^. Many other species first detected by DNA differences have retrospectively been shown to be morphologically distinguishable, representing cases of cryptic or morphologically closely similar species^33^,^34^,^35^,^36^,^37^,^38^,^39^,^40^,^41^,^42^ (whether a species is similar enough to be ‘cryptic’ or not is a fine line). A remarkable amount of DNA barcode data has been generated during recent years in association with the campaigns of the International Barcode of Life Project (iBOL, http://ibol.org/). While it is evident that massive barcoding campaigns of regional biotas will further accelerate species discoveries especially in poorly investigated areas and groups, several case studies have demonstrated unexpected discoveries among characteristic species and in well-known regions^34^,^36^,^37^.

We used moths of the genus *Elachista* (Lepidoptera: Gelechioidea: Elachistidae) as an exemplar group. The genus worldwide comprises approximately 700 named species^43^. We targeted the *Elachista bifasciella* group of the Palearctic region. These are difficult to identify. This is because of the large number of species (130 species described worldwide), small size (wingspan typically 5–12 mm), drab coloration, generally little differentiation in external morphology, and subtle differences in genitalia (Figure 1, where a maximum of variation in appearance and morphology within the group is shown). They have nevertheless been the subject of thorough morphology-based taxonomic investigations during the past decades^44^,^45^,^46^,^47^,^48^,^49^,^50^,^51^,^52^,^53^,^54^,^55^,^56^ covering the fauna of the whole Palearctic region, although the European part of the region is evidently better investigated than that of other areas. As species of *E. bifasciella* group show close similarity in their morphology, an assumption that the species have diverged quite recently seems plausible. DNA barcodes are expected to show low resolution in such circumstances, because loci of young species may not have had sufficient time to fully diverge (often called incomplete lineage sorting) and may have been subjected to mitochondrial introgression^57^.

We examined the performance of COI barcodes in differentiating morphologically similar and allegedly young species of the Palearctic *bifasciella* group. We also examined whether broad sampling of collection specimens would reveal genetically distinct lineages, potentially representing new species. We went still further and examined whether these genetically distinct lineages bear unique morphological characteristics that would further support their validity as new species. While large-scale analyses of DNA barcodes have often documented intraspecific genetic splits^10^,^30^,^31^,^58^, few of them have been extended to systematically examine whether such lineages represent entities that gain integral support by independent lines of evidence, such as nuclear DNA, morphology or life-history traits, although there are notable exceptions^30^,^59^,^60^). We used morphology-based research of the group as a backbone hypothesis, without making presumptions as to whether they are correct in all details.

## Results

The original analysis of data revealed several species having considerable (>1%) intraspecific variation and likewise clusters of unidentified specimens showing a distinct gap (>1%) from any pre-identified specimens. Altogether, twenty-five such cases were detected. All these were subjected to an in-depth morphological examination. This resulted in the detection of sixteen putative new species whose species integrity was independently supported by genetic and morphological uniqueness. Eight of these species were from Russia, three from Kyrgyzstan, two from Greece and one each from Slovenia, Turkey and Ukraine (Figure S1). Formal descriptions of these taxa have been prepared and will be published in another context (L. Kaila, in prep). Detailed morphological diagnoses for the new species will be provided in a forthcoming paper. A summary of key diagnostic features with regards of all other species is presented in Table S1. In a few cases of species showing a remarkable (>1%) intraspecific split, notably *E. maculicerusella* and *E. canapennella*, the morphological examination failed to support the presence of multiple distinct lineages.

The mean of minimum K2P distances between the genetically closest species, with both described and newly discovered putative species included, was 3.82% (range 0%–7.41%) (Figure 2, Table 1). The same measure was 3.98% (range 0–7.41%) and 3.42% (range 1.08–6.9%) in the described species and putative new species, respectively. The maximum intraspecific variation across species (singletons excluded, newly discovered putative species included) ranged from 0% to 3.76%, with an average across the species of 0.74% in described species (35 species) and 0.61% with newly discovered species included (45 species). The maximum intraspecific variation exceeded 1% in 10 species and 2% in 4 species.

BIN algorithms split the data into 49 distinct categories (Figure S2). In two cases, a single species contained two BINs (*E. maculicerusella*, *E. canapennella*), while five BINs contained two species, and two BINs three species. Of the 16 newly detected putative species, 11 had unique BINs, whereas 5 newly detected putative species, although having clearly distinct barcodes, were included in a BIN containing one or two additional species.

## Discussion

**Figure 1:**
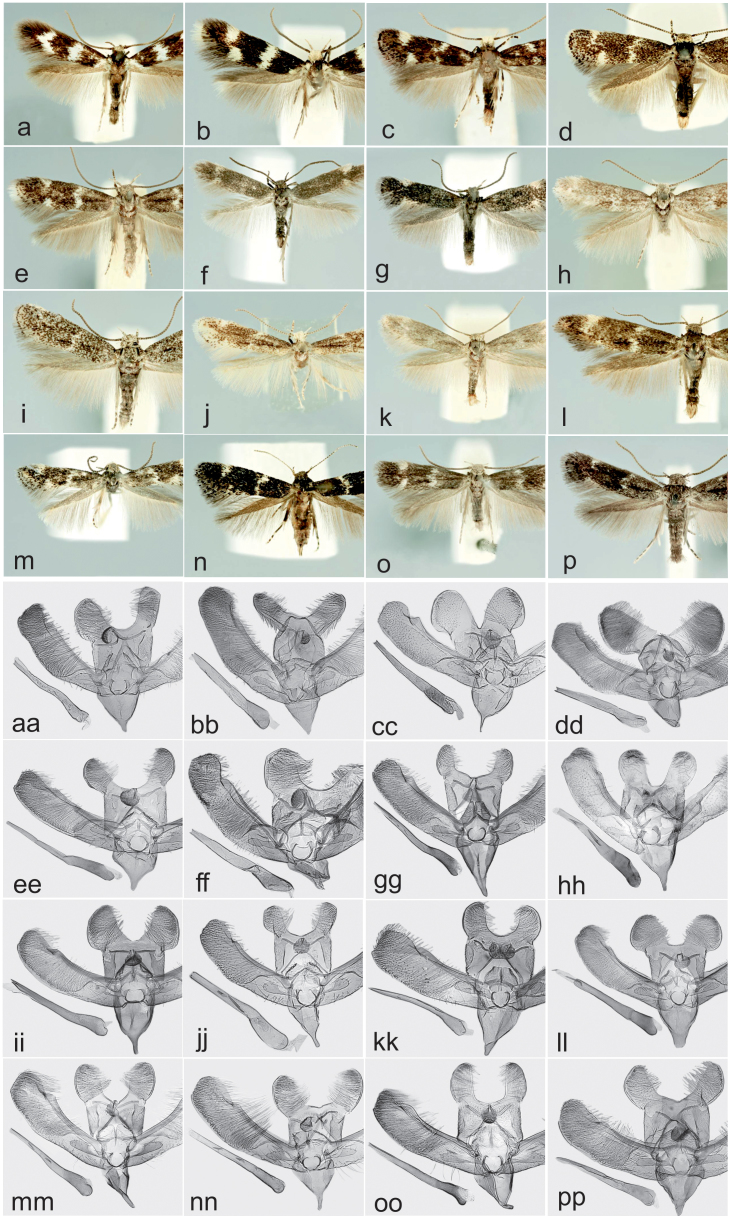
Habitus and male genitalia of selected N. European species of *Elachista bifasciella* group (habitus in comparable scale). (a), aa: *E. bifasciella*; (b), bb: *E. dimicatella*; (c), cc: *E. albifrontella*; (d), dd: *E. griseella*; (e), ee: *E. kilmunella*; (f), ff: *E. irenae*; (g), gg: *E. humilis*; (h), hh: *E. herrichii*; (i), ii: *E. orstadii*; (j), jj: *E. canapennella*; (k), kk: *E. krogeri*; (l), ll: *E. atricomella*; (m), mm: *E. deriventa*; (n), nn: *E. elegans*; (o), oo: *E. nielswolffi*; (p), pp: *E. poae*. The photographs were taken by LK.

**Figure 2:**
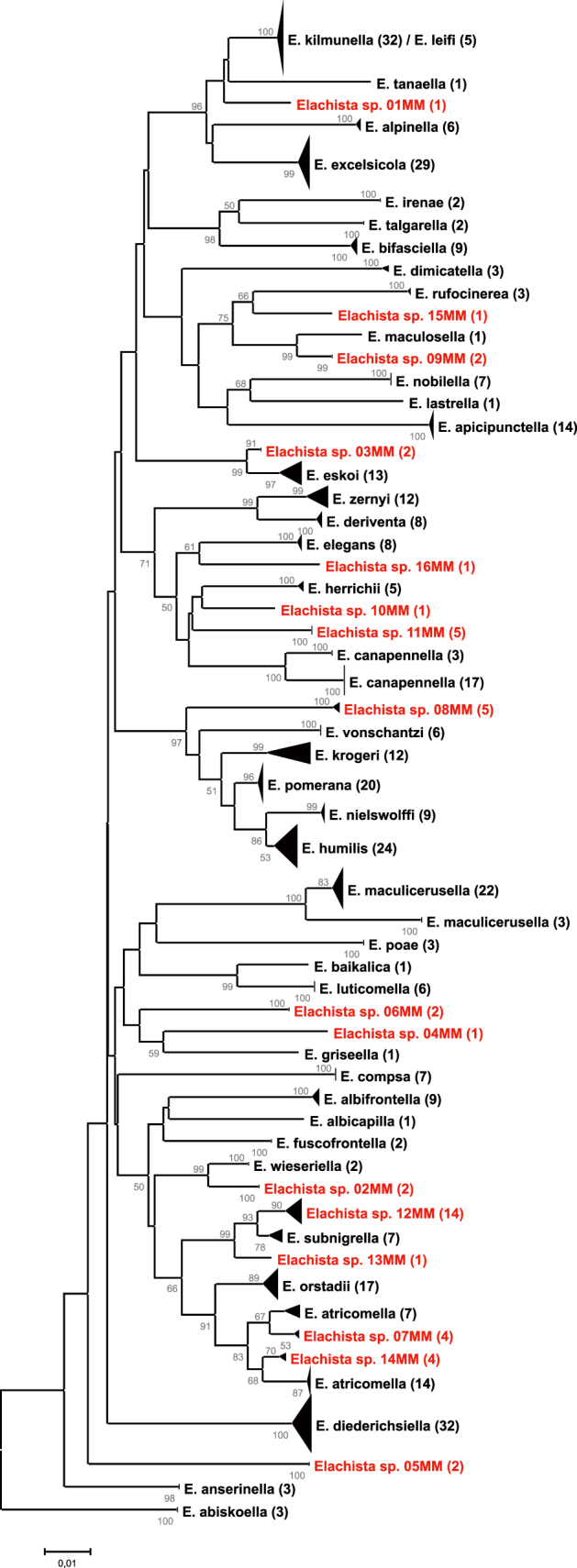
A Neighbor-Joining tree, generated under the K2P nucleotide substitution model, of the study taxa. Node bootstrap support values of >50% are indicated. Sample size for each species is indicated in parentheses after species names. The height of terminal triangles is proportional to sample size, the depth to degree of intraspecific variation. Deep (>2%) intraspecific splits are shown separated. Newly discovered species are highlighted in red.

Our study provides insights into the two fundamental features of DNA barcoding. First, the study serves as a test of the utility of DNA barcodes in recognizing species. Second, we show that broad sampling of museum specimens aids the discovery of new species in a remarkable way. Species of the test group belong to a diverse group of moths that include species that generally have overall similar morphology (Figure 1), but which have been under unusually comprehensive morphological taxonomic scrutiny. The former factor is likely to increase the proportion of previously undetected species, while the latter is expected to show the reverse effect. Provided that few groups of small-sized moths, let alone most other insect groups, have been under taxonomic scrutiny comparable to that of *Elachista* of the study region, we assume that surveys conducted with other insect groups would similarly lead to the discovery of many new species. Some former large-scale investigations have similarly yielded the discovery of several apparent new species^61^,^62^.

Based initially on gaps in genetic variation of DNA barcode regions, we discovered twenty-five lineages that were either not closely associated with identified specimens or showed considerable (>1%) intraspecific variation. Fourteen of these cases were subsequently shown to represent two distinct lineages and one other (*E. atricomella*) three distinct lineages, as demonstrated by independent sources of evidence, notably differences in external appearance and in genital structures, which generally evolve rapidly and divergently, hence providing cues for the separation of young species^63^. Following traditionally used criteria of species delimitation in the study group and insect taxonomy in general, these lineages can be seen to represent valid species. However, species delineation is inherently subjective as it depends, for example, on the species concept applied. Two additional species are genetically split into two widely different clusters and separate BINs. One of these splits (*E. maculicerusella*) concern sympatric populations and has previously been suspected of comprising two species based on possibly differing external appearance and life history traits, while in the second case (*E. canapennella*) the split is correlated with a wide geographic gap. Whether these species truly contain cryptic diversity should preferably be examined with other lines of evidence. As we were not able to find constant morphological differences between these clusters, the sequencing of nuclear markers might provide further insights into the question (cf.^40^). Mitochondrial and nuclear genomes evolve independently, and different nuclear genes may vary in their gene genealogies. Therefore, nuclear genes have a great potential for resolving whether mitochondrial haplotypes represent either panmictic populations or truly separate, reproductively isolated lineages. This is most applicable when the taxa in question occur in sympatry, because in such circumstances nuclear genes are not expected to show consistent differences as compared to mitochondrial haplogroups within a species.

The Lepidoptera in the study area have been much more intensively studied than in any other area of the world, suggesting that a similar survey conducted elsewhere with a comparable effort would reveal even more new species. Furthermore, while our survey increased the number of known species by one-third, we recognize that a still broader sampling of material from collections would almost inevitably lead to the discovery of still more undetected species. Our sample comprised only a small fraction of specimens deposited in zoological museums and private collections and does not include samples from wide areas, including sites known to host endemic taxa in other insect groups. Although the new species were retrospectively observed to be unique in their morphology, we are convinced that several of them would have remained undetected for some time without DNA barcodes. Numerous recent taxonomic studies have demonstrated the usefulness of DNA barcoding in initial detection of taxa that have been recognized as new after closer examination^33^,^34^,^35^,^36^,^37^,^38^,^39^,^40^,^41^,^42^. Our study is rare in the breadth of sampling and the independent validation of genetic results with morphological evidence, including the large-scale examination of genital structures. An example of a different pattern is reported in a study including an in-depth morphological examination of eight genetically highly variable Romanian butterfly species. This study did not yield evidence for any additional species^30^. European butterflies are, however, an exception among all insect groups in the extent of previous morphology-based investigation and hence generally lower levels of new species discoveries are expected in this group than in most other insect groups.

All previously named species, except *E. kilmunella* and *E. leifi* which share the same haplotypes, could unequivocally be identified based on barcodes alone, equating to 95% identification success. This provides evidence not only of the efficiency of DNA barcodes in separating species, but also of the general validity of traditionally used criteria for delimiting species in the study group. The validity of *E. leifi* has been under dispute, as it shows only minor morphological differences to *E. kilmunella*^47^. *E. leifi* males swarm only at dawn, while *E. kilmunella* males are day- and dusk-active. There is yet the possibility that *E. leifi* is a northern, yet sympatric form of *E. kilmunella*, perhaps adapted to a two-year life cycle, a phenomenon known from many lepidopteran species. This alternative does not, however, explain the behavioural difference. Genetic identifiers may, however, also fail to discriminate species. Especially with young species, the time elapsed since speciation may have not been sufficient for lineage sorting to be completed^16^,^61^. Young species may also show introgression^16^. We tested the utility of DNA barcodes in multiple cases of conditions where both of those phenomena were anticipated to occur, but found the pair *E. kilmunella*/*E. leifi* to be the only one in which these effects are possibly involved.

*E. atricomella* was found to constitute three genetically and morphologically distinct species, two of which were given a status of putative new species. This split rendered the remaining specimens of *E. atricomella* as forming a polyphyletic species. While incomplete lineage sorting or introgression may result in gene-level para- or polyphyly, we cannot rule out that this species includes a fourth, morphologically indistinguishable species, pending the examination of other genetic markers.

Two or three species were lumped together by BIN algorithms in seven cases. In each of these, except with *E. kilmunella* and *E. leifi*, the ability of DNA barcodes to discriminate species remained. BINs are deliberately designed to be relatively conservative in highlighting species having high potential of cryptic diversity and in minimizing the over-splitting of species^62^. We consider the conservativeness of BINs to be a meaningful property, as the unjustified splitting of species with accumulated synonyms is seen as having a more confusing impact in taxonomy than the opposite.

In summary, our results support many earlier observations that DNA barcodes effectively differentiate closely related species. We demonstrate that a comprehensive sampling of collection material is an efficient way to discover hidden portions of biodiversity and that by accelerating taxonomic workflow DNA barcoding provides an important tool that, when widely used, might substantially help to overcome the taxonomic impediment. Lepidoptera represents one of the most thoroughly investigated groups of insects, and our focal group has been under considerable previous taxonomic investigation. Our results therefore suggest that the assessment of insect species number may often be underestimated by the overlooking of morphologically similar species. Along with growing DNA barcoding activity, we assume an increasing rate of discoveries of new species across all insect groups and areas.

## Methods

We aimed at sampling all Palearctic species of the *E. bifasciella* group, excluding those of Japan and the Russian Far East (Sakhalin, Primorsk Region), from where we could not obtain samples. A total of 49 named species are known from this region. We obtained 40 of these for study (Table S2). Besides a focused sampling of identified specimens of described species, we performed a bulk sampling of unidentified museum and other collection specimens, paying attention particularly to samples from areas whose fauna has been less exhaustively studied. Our sampling is geographically biased towards northern Europe, where 31 out of 40 sampled species occur (of these only *E. herrichii* was barcoded outside this region). We supplemented this sampling with available fresh specimens from Central and South Europe, from the Ural region and several areas of Central Asia and eastern Siberia. Given the vast area of the Palearctic region, our sampling cannot be considered exhaustive in geographic terms. Similarly, in most species specimens were not comprehensively sampled from different areas of their known range. Localities for the specimens included in this study are indicated in the Figure S1. The map was created using an online tool SimpleMappr^64^.

All specimens subjected to DNA sequencing were given a label with a unique sample ID. One or two legs of each specimen were deposited in microplate wells with 30 μL of absolute alcohol in each well. The sequencing was carried out at the Canadian Centre for DNA Barcoding following laboratory protocols used routinely in the Canadian Centre for DNA Barcoding (CCDB) as explained in detail in DeWaard *et al.*^65^. In short, this involves the Chelax-based Dry-Release DNA extraction done for an aliquot of 30–110 μL. A volume of 0.5–2 μL of aliquot is used for the PCR. CCDB uses a wide set of PCR primers for DNA amplification, depending, e.g., on the taxon group and specimen condition. The primers used, as with all other laboratory protocol details, are available at the sequence page of each record at the BOLD database. In Lepidoptera, the primer pair used is usually LepF1-LepR1. PCR is done in a volume of about 11–13 μL. PCR products are checked using Invitrogen E-gel 96 system with precast and bufferless agarose gels. Since sequencing is done bi-directionally, the PCD-products are not purified, but sequencing reactions are set up directly from PCR products. Depending on the intensity of gel bands, 0.5–2 μL of PCR product is used for sequencing reactions. The sequencing follows a routinely used recipe of ingredients with Dye terminator mix v3.1 and each reaction is done in a volume of 10 μL. Sequence reaction cleanup is done using the Sephadex column method. Sequence alignments are done using programs (Sequencer, SeqScape, Lasergene) permitting the assembling of bidirectional reads and trace file edits. Sequences were carefully checked for the detection of COI pseudogenes (NUMTS) and contaminations. Consequently, a sample of *E. anserinella* contaminated by another species was discarded.

A total of 437 specimens yielded at least a partial barcode sequence, with 95% of the barcodes being over 600 bp in length (Table S3). In only six cases were barcodes of less than 400 bp included, none of which were the sole representative of their species. The average sample size per species with sixteen discovered putative new species assessed as separate species was 7.8 (range 1–32). Distance statistics were calculated using BOLD (Barcode of Life Data Systems) tools, accessible at http://www.boldsystems.org^66^. The Barcode Index Number (BIN) grouping of haplotypes into Operational Taxonomic Units (OTUs)^62^, a recent option of BOLD, was used in the interpretation of results. The BIN delimitation of sequences into OTUs includes two steps. In the first stage, sequences are clustered based on the single linkage clustering method. In the second step, this preliminary clustering is refined using graph-based Markov Clustering (MCL). Based on eight independent datasets, BINs were shown to have very high correspondence with pre-existing assignments of species^62^. In this study, we used BIN assignments only for advisory purposes. We did not base our species delimitation on them nor on other quantitative delimitation algorithms, because in addition to molecular data, we incorporated morphological, though not quantitative morphometric data, in our analyses.

Sequences were aligned using BOLD alignment, which performs the alignment against a wide variety of animal barcodes deposited in BOLD. A few resulting gaps were subsequently removed. Sequence alignments were manually checked in Mega 5^67^, resulting in the detection of a misalignment of a single codon in two cases. A Neighbor-Joining tree was constructed using Mega 5 under the Kimura 2 Parameter (K2P) model, with pairwise deletion of missing data and 500 bootstrap pseudoreplicates in order to test node robustness, especially for nodes leading to species. The full specimen collection details, voucher photographs, sequence data with trace files and GenBank accession numbers are available under the public dataset DS-ELABIF in BOLD. Information on specimens is also provided in the Table S3.

All species showing clear discontinuities (over 1% intraspecific gap; also including one case having a 0.99% gap) or an unusual extent of more continuous intraspecific variation (over 1% maximum intraspecific variation) were subjected to an in-depth morphological examination. Likewise, specimens not directly linked to any named taxa were examined morphologically. Morphological examination was based primarily on male and female genital characteristics, but also wing patterns were examined. This follows traditional guidelines of species delimitation in *Elachista*, where typically slight or moderate differences in genitalia have been used as criteria in species delimitation. This seems generally well-justified as in well-investigated species morphological delimitation criteria have been shown to correlate with different life-history properties, such as larval food plants. Species of the study group typically show narrow diets and high level of niche specialization.

For the study of the morphology of the genitalia, the abdomen was macerated in 10% aqueous solution of potassium hydroxide, rinsed thoroughly in clean water, then put to an object slide where the water was replaced by absolute EtOH. Excessive scales were removed to improve clarity. The abdominal pelt, as well as the female genitalia, were stained using chlorazol black, and the male genitalia using yellow eosin. The genitalia were severed from the abdomen by cutting the abdomen along the pleurum and along intersegment 7 and 8. The abdomen and the genitalia were transferred to another slide, to which a drop of Euparal™ was applied. In the male genitalia, the phallus was gently severed from the genital capsule. The valvae were gently opened, and the uncus, vinculum and valvae were slightly pressed using forceps to align them in the horizontal plane, and the phallus positioned in a lateral position, sometimes dorsoventrally, so as to better allow the examination of the shape of the often bifurcate apex. The genitalia were then covered by a cover glass and incubated in 40°C for two weeks. The female genitalia were positioned ventral-side upwards.

The delimitation of species in Elachistidae, as usually in animal taxonomy, assumes that the species are ‘natural’ units, the underlying hypothesis being that the species are populations or clusters of populations that may interbreed in nature, i.e., they form cohesive genealogical units. This is, of course, next to impossible to directly observe. Therefore indirect information, derived from morphology and life history traits, is routinely used for obtaining an approximation of species delimitation. Each ‘species’ recognized is therefore a hypothesis that can, and should, be subjected to further testing. The most obvious methods for testing are comparing samples collected in ‘new’ localities, acquiring further biological knowledge by rearing of larvae, and studying genomic traits (cf.^43^)

As criteria for the evaluation of possible morphological distinction, special attention was paid to the following traits, following the general tradition, and morphological particulars of the focal group (terminology follows Traugott-Olsen & Schmidt Nielsen 1977^45^ and Kaila 1999^53^): in external appearance, the wing pattern and shape, the thickness of antennae and length of the labial palpi, and the colour of the head and the neck tuft; in male genitalia, the relative size and shape of the uncus, gnathos, valva, digitate process, phallus and cornuti; in the female genitalia, the size, shape and position of the ostium bursae, the shape of the antrum, the inception position and shape of the ductus seminalis, the length and sclerotisations of the ductus bursae, the shape of the corpus bursae, and the size and shape of the signum.

## Author Contributions

M.M. and L.K. designed the study. All authors took part in material acquisition and preparing samples for DNA analyses. M.M. performed analyses of molecular data and L.K. analysed morphological data. M.M. and L.K. prepared the figures and tables. M.M and L.K. wrote the manuscript and J.T. commented it.

## Supplementary Material

Supplementary Information Supplementary information

## Figures and Tables

**Table 1:** Distance statistics (numbers in per cents, implemented under K2P nucleotide substitution model) of *Elachista bifasciella* group of species with observed putative species included in the comparison

Species	Mean intraspecific	Max intraspecific	Nearest species	Min distance to Nearest Species
Elachista albicapilla	N/A	N/A	Elachista orstadii	4.53
Elachista albifrontella	0.2	0.31	Elachista orstadii	5.59
Elachista alpinella	0.1	0.32	Elachista sp. 01 MM	4.45
Elachista anserinella	0	0	Elachista orstadii	4.52
Elachista apicipunctella	0.02	0.15	Elachista sp. 09 MM	7.23
Elachista atricomella	1.2	2.7	Elachista sp. 07 MM	1.08
Elachista baikalica	N/A	N/A	Elachista luticomella	3.15
Elachista bifasciella	0.14	0.32	Elachista talgarella	5.85
Elachista canapennella	0.63	2.41	Elachista elegans	5.4
Elachista compsa	0	0	Elachista pomerana	6.94
Elachista deriventa	0.12	0.32	Elachista zernyi	2.13
Elachista diederichsiella	0.47	1.4	Elachista orstadii	5.65
Elachista dimicatella	0.21	0.24	Elachista nobilella	6.75
Elachista elegans	0.07	0.15	Elachista sp. 16 MM	4.94
Elachista eskoi	0.38	1.08	Elachista sp. 03 MM	1.39
Elachista excelsicola	0.23	1.24	Elachista kilmunella	3.62
Elachista fuscofrontella	0	0	Elachista sp. 02 MM	4.72
Elachista griseella	N/A	N/A	Elachista sp. 14 MM	6.39
Elachista herrichii	0.12	0.31	Elachista sp. 10 MM	3.78
Elachista humilis	0.52	1.4	Elachista nielswolffi	1.01
Elachista irenae	0	0	Elachista talgarella	5.88
Elachista kilmunella	0.18	0.92	Elachista leifi	0
Elachista krogeri	0.55	1.97	Elachista pomerana	2.68
Elachista lastrella	N/A	N/A	Elachista nobilella	6.57
Elachista leifi	0	0	Elachista kilmunella	0
Elachista luticomella	0	0	Elachista baikalica	3.15
Elachista maculicerusella	0.94	5.29	Elachista lastrella	7.1
Elachista maculosella	N/A	N/A	Elachista sp. 09 MM	2.18
Elachista nielswolffi	0.07	0.25	Elachista humilis	1.01
Elachista nobilella	0	0	Elachista lastrella	6.57
Elachista orstadii	0.34	0.77	Elachista atricomella	2.06
Elachista poae	0	0	Elachista sp. 14 MM	7.41
Elachista pomerana	0.08	0.31	Elachista humilis	1.56
Elachista rufocinerea	0.05	0.15	Elachista sp. 15 MM	4.75
Elachista subnigrella	0.27	0.61	Elachista sp. 12 MM	1.24
Elachista talgarella	0	0	Elachista bifasciella	5.85
Elachista tanaella	N/A	N/A	Elachista kilmunella	4.17
Elachista vonschantzi	0	0	Elachista pomerana	3.59
Elachista wieseriella	0	0	Elachista sp. 02 MM	2.02
Elachista zernyi	0.4	1.26	Elachista deriventa	2.13
Elachista sp. 01 MM	N/A	N/A	Elachista kilmunella	2.66
Elachista sp. 02 MM	0	0	Elachista wieseriella	2.02
Elachista sp. 03 MM	0	0	Elachista eskoi	1.39
Elachista sp. 04 MM	N/A	N/A	Elachista griseella	6.53
Elachista sp. 05 MM	0	0	Elachista anserinella	6.9
Elachista sp. 06 MM	0	0	Elachista fuscofrontella	6.56
Elachista sp. 07 MM	0.21	0.41	Elachista atricomella	1.08
Elachista sp. 08 MM	0.15	0.31	Elachista humilis	3.62
Elachista sp. 09 MM	0	0	Elachista maculosella	2.18
Elachista sp. 10 MM	N/A	N/A	Elachista herrichii	3.78
Elachista sp. 11 MM	0	0	Elachista herrichii	4.44
Elachista sp. 12 MM	0.33	1.24	Elachista subnigrella	1.24
Elachista sp. 13 MM	N/A	N/A	Elachista subnigrella	1.87
Elachista sp. 14 MM	0.12	0.41	Elachista atricomella	1.39
Elachista sp. 15 MM	N/A	N/A	Elachista sp. 09 MM	4.14
Elachista sp. 16 MM	N/A	N/A	Elachista elegans	4.94
