# Supplementary material for: Wide-ranging barcoding aids discovery of one-third increase of species richness in presumably well-investigated moths
Source: Sci Rep. 2013 Oct 9;3:2901. doi: 10.1038/srep02901 (PMC3793226; doi:10.1038/srep02901)
Supplement: Supplementary Information [file srep02901-s1.pdf]

Supplementary information

Wide-ranging barcoding aids discovery of one-third increase of species richness in presumably well-investigated moths

Marko Mutanen, Lauri Kaila & Jukka Tabell

Table S1. Diagnostic features of the putative new species recognized in this study. Characters given are short summaries of features that best distinguish them from their most similar relatives. Comprehensive diagnoses and descriptions, beyond the scope of this publication, will be published separately by L. Kaila (in prep). Number of morphologically examined specimens is indicated for each species in parentheses after the interim name.

**Interim name for a putative species**	**Diagnostic characteristics**
***Elachista* sp. 01MM (1)**	Forewing: nearly unicolorous, shiny dark grey<br>Male genitalia: large uncus, large gnathos, long and broad valva
***Elachista* sp. 02MM (2)**	Forewing: narrow, shiny pale grey with costal and tornal spots opposite to each other<br>Male genitalia: very large uncus lobes, very small spinose knob of the gnathos
***Elachista* sp. 03MM (2)**	Forewing: dark grey, fascia extends to costa<br>Male genitalia: very small gnathos
***Elachista* sp. 04MM (1)**	Forewing: narrow, two white fasciae on grey ground colour<br>Male genitalia: small uncus lobes, long saccus
***Elachista* sp. 05MM (5)**	Forewing: unicolorous, creamy-coloured<br>Male genitalia: narrowly bifurcate apex of phallus, distally rounded juxta lobes
***Elachista* sp. 06MM (12)**	Forewing: uniquely narrow; pale yellow<br>Male genitalia: broad gnathos, apex of phallus straight-cut
***Elachista* sp. 07MM (56)**	Small, stout-bodied. Forewing: ground colour black; costal and tornal spots white, separate.<br>Male genitalia: as *E. atricomella.*<br>Female genitalia: posterior margin of sternum 7 strongly curved; spines of antrum very coarse
***Elachista* sp. 08MM (5)**	Forewing: dark grey with three white markings, indistinct in male, distinct in female<br>Male genitalia: very broad valva, elongate uncus; female: narrow ostium, short antrum
***Elachista* sp. 09MM (2)**	Forewing: pale yellow, indistinct brown irroration<br>Male genitalia: broad apex of phallus; broad digitate process.<br>Female genitalia: entirely sclerotized papillae anales; specific shape of antrum
***Elachista* sp. 10MM (1)**	Small. Forewing: ground colour pale brown; antenna: annulated with white rings<br>Male genitalia: uncus lobes large and rounded; two cornuti of equal size
***Elachista* sp. 11MM (5)**	Forewing: dark grey with three bright white markings<br>Male genitalia: small uncus lobes, long and distally widened valva; apex of phallus not bifurcate<br>Female genitalia: ostium deeply incised in sternum 7; antrum short
***Elachista* sp. 12MM (21)**	Large. Forewing: dark grey with distinct white markings; shape elongate<br>Male genitalia: valva narrow and long, not broadened distally; large cornutus<br>Female genitalia: large and elongate antrum with coarse setae; inception of ductus seminalis close to antrum; base of ductus seminalis dilated
***Elachista* sp. 13MM (6)**	Small.Forewing: unicolorous, mottled dark grey<br>Male genitalia: valva not bent; small cornutus, small uncus lobes
***Elachista* sp. 14MM (15)**	Large. Forewing: ground colour suffused, grey; costal and tornal spots confluent.<br>Male genitalia: as *E. atricomella.*<br>Female genitalia: posterior margin of sternum 7 straight
***Elachista* sp. 15MM (4)**	Forewing: mottled pale grey with confluent costal and tornal spots, dark grey spot at dorsum<br>Male genitalia: uncus lobes elongate; caecum absent
***Elachista* sp. 16MM (1)**	Forewing: mottled dark grey with irregularly shaped pale median area<br>Male genitalia: narrow phallus with one cornutus; uncus lobes proximate

Table S2. Presently named Eurosiberian species of *bifasciella* group (47 spp.). The area considered covers Palearctic region excluding Japan and Russia: Sakhalin and the Primorsk region. Himalaya is also excluded. From North Africa, also a part of the Palearctic region, no species of *E. bifasciella* group has been reported.

Elachista adelpha Kaila & Jalava, 1994
Elachista albicapilla Höfner, 1918
Elachista albifrontella (Hübner, (1817)
Elachista alpinella Stainton, 1854
Elachista anserinella Zeller, 1839
Elachista anserinelloides Nel, 2003
Elachista apicipunctella Stainton, 1849
Elachista argentifasciella Höfner, 1898
Elachista atricomella Stainton, 1849
Elachista baikalica Kaila, 1992
Elachista bicingulella Sruoga, 1992
Elachista bifasciella Treitschke, 1833
Elachista canapennella (Hübner, 1813)
Elachista compsa Traugott-Olsen, 1974
Elachista deriventa Kaila & Mutanen, 2008
Elachista diederichsiella Hering, 1889
Elachista dimicatella Rebel, 1903
Elachista elegans Frey, 1859
Elachista eskoi Kyrki & Karvonen, 1985
Elachista excelsicola Braun, 1948
Elachista fuscofrontella Sruoga, 1990
Elachista grandiferella Sruoga, 1992
Elachista griseella (Duponchel, 1843)
Elachista gruenewaldi Parenti, 2002
Elachista herrichii Frey, 1859
Elachista humilis Zeller, 1850
Elachista irenae Buszko, 1989
Elachista jaskai Kaila, 1998
Elachista kilmunella Stainton, 1849
Elachista krogeri Svensson, 1976
Elachista lastrella Chrétien, 1896
Elachista leifi Kaila & Kerppola, 1992
Elachista luticomella Zeller, 1839
Elachista maculicerusella (Bruand, 1859)
Elachista maculosella Chrétien, 1896
Elachista nielswolffi Svensson, 1976
Elachista nobilella Zeller, 1839
Elachista orstadii Palm, 1943
Elachista poae Stainton, 1855

Elachista pomerana Frey, 1870
Elachista rufocinerea (Haworth, 1828)
Elachista stenopterella Rebel, 1932
Elachista subnigrella Douglas, 1853
Elachista sulcsiella Savenkov, 2013
Elachista talgarella Kaila, 1992
Elachista tanaella Aarvik & Berggren, 2004
Elachista vonschantzi Svensson, 1976
Elachista wieseriella Huemer, 2000
Elachista zernyi Hartig, 1941

Table S3. Specimen information.

Sample ID	Species	Seq. Length	Sex	Collectors	Collection Date	Country	Locality	Lat	Lon	Elev	Notes
MM17678	Elachista abiskoella	657[1n]		Erkki Laasonen, Leena Laasonen	01-Jul-2001	Finland	Lapponia inarensis, Inari	68,39	28,22		
MM18594	Elachista abiskoella	658		Bengt A. Bengtsson	02-Jul-2004	Sweden	To, Abisko, Oestra	68,30	18,70		
MM19891	Elachista abiskoella	658	M	Erkki Laasonen, Leena Laasonen		Finland	Lapponia inarensis, Inari	68,47	28,28		
MM20832	Elachista albicapilla	658	M	P. Huemer	06-Aug-1996	France	Alpes-Maritimes, Marguareis W-Hang, Navela	44,16	7,66	2150	
MM02662	Elachista albifrontella	657[1n]		Marko Mutanen, Panu Vaelimaeki		Finland	Karelia australis, Imatra	61,11	28,80		
MM06161	Elachista albifrontella	658		Marko Mutanen, Tomi Mutanen	18.-17.6.2007	Finland	Karelia ladogensis, Parikkala	61,64	29,64		
MM06514	Elachista albifrontella	658	M	Marko Mutanen, Nestori Mutanen	03-Jul-2007	Finland	Ostrobottnia borealis pars australis, Hailuoto	65,05	24,88		
MM06598	Elachista albifrontella	658	F	Tomi Mutanen, Marko Mutanen, Nestori Mutanen, Anttoni Mutanen	6.-7.7.2007	Finland	Karelia borealis, Ilomantsi	62,51	30,88		
MM12029	Elachista albifrontella	658		Marko Mutanen, Panu Vaelimaeki	2007	Finland	Nylandia, Hanko	59,84	23,24		
MM14235	Elachista albifrontella	658		Marko Mutanen, Nestori Mutanen, Anttoni Mutanen	29-Jun-2009	Finland	Aland Islands, Eckerö	60,24	19,59		
MM14309	Elachista albifrontella	658		family Marko Mutanen	30-Jun-2009	Finland	Aland Islands, Finstroem	60,23	19,83		
MM20887	Elachista albifrontella	658	M	T. Nupponen, K. Nupponen	24-Jun-2000	Russia	Altai Krai, Altai Mts., Sarlyk region	51,00	85,58	1450	
MM20891	Elachista albifrontella	658	M	T. Nupponen, K. Nupponen	23-Jun-2000	Russia	Altai Krai, Altai Mts., Katun valley, 10 km SE Ust-Sema vill.	51,58	85,92	700	
MM03989	Elachista alpinella	639		Marko Mutanen	14-Jul-2006	Finland	Ostrobottnia borealis pars australis, Kiiminki	65,07	25,73		
MM06929	Elachista alpinella	658	M	Marko Mutanen, Nestori Mutanen, Anttoni Mutanen	26-Jul-2007	Finland	Ostrobottnia borealis pars australis, Kiiminki	65,07	25,73		
MM10427	Elachista alpinella	658		Marko Mutanen	15-Jul-2004	Finland	Ostrobottnia borealis pars australis, Kiiminki	65,07	25,73		
MM12473	Elachista alpinella	658		Marko Mutanen, Panu Vaelimaeki	2007	Finland	Savonia australis, Imatra	61,11	28,80		
MM16180	Elachista alpinella	658	M	Jalava, Kullberg, Koponen	08-Jul-1994	Russia	Polar Ural, Krasnyi Kamen	66,08	65,17		L.Kaila prep. 1373
MM21196	Elachista alpinella	658		Marko Mutanen	10-Jul-2011	Finland	Regio kuusamoensis, Kuusamo	66,38	29,43		
MM18556	Elachista anserinella	658		Ingvar Svensson	06-Jun-2006	Sweden	Gotland, Loista	57,31	18,36		
MM19870	Elachista anserinella	407		N. Savenkov, I. Savenkov	09-Jun-2006	Sweden	Gotland, Silte	57,21	18,23		

MM21442	Elachista anserinella	658	M	N. Savenkov	17-May-2010	Bulgaria	Pirin, Sandanski Ploski	41,64	23,26	250	
MM02659	Elachista apicipunctella	658		Marko Mutanen, Panu Vaelimaeki		Finland	Karelia australis, Imatra	61,11	28,80		
MM02660	Elachista apicipunctella	658		Marko Mutanen, Panu Vaelimaeki		Finland	Karelia australis, Imatra	61,11	28,80		
MM06170	Elachista apicipunctella	658		Marko Mutanen, Tomi Mutanen	18.-17.6.2007	Finland	Karelia ladogensis, Parikkala	61,64	29,64		
MM06173	Elachista apicipunctella	658		Marko Mutanen, Tomi Mutanen	18.-17.6.2007	Finland	Karelia ladogensis, Parikkala	61,64	29,64		
MM06225	Elachista apicipunctella	658		Marko Mutanen	19-Jun-2007	Finland	Karelia australis, Joutseno	61,06	28,72		
MM06271	Elachista apicipunctella	658	M	Marko Mutanen, Nestori Mutanen, Anttoni Mutanen	22.-23.6.2007	Finland	Ostrobottnia borealis pars australis, Kiiminki	65,07	25,73		
MM06272	Elachista apicipunctella	658	F	Marko Mutanen, Nestori Mutanen, Anttoni Mutanen	22.-23.6.2007	Finland	Ostrobottnia borealis pars australis, Kiiminki	65,07	25,73		
MM08382	Elachista apicipunctella	658		Marko Mutanen, Panu Vaelimaeki	17.-18.6.2005	Finland	Tavastia australis, Loppi	60,65	24,08		
MM09050	Elachista apicipunctella	658		Marko Mutanen, Panu Vaelimaeki	2006	Finland	Karelia ladogensis, Simpele	61,42	29,32		
MM14139	Elachista apicipunctella	658		Marko Mutanen, Nestori Mutanen, Anttoni Mutanen, Rosella Mutanen	19-Jun-2009	Finland	Ostrobottnia borealis pars australis, Kiiminki	65,07	25,73		
MM14208	Elachista apicipunctella	658		MarkoMutanen	24-Jun-2009	Finland	Ostrobottnia borealis pars australis, Oulu	64,94	25,46		
MM14270	Elachista apicipunctella	658		family Marko Mutanen	30-Jun-2009	Finland	Aland Islands, Geta	60,33	19,91		
MM20930	Elachista apicipunctella	658	M	K. Nupponen	17-Jun-2002	Russia	Irkutsk, Irkutsk prov., Sludjanka village 2 km SE, Lake Baikal shope slope	51,67	103,7 2	500	
MM20935	Elachista apicipunctella	658	M	K. Nupponen	17-Jun-2002	Russia	Irkutsk, Irkutsk prov., Sludjanka village 2 km SE, Lake Baikal shope slope	51,67	103,7 2	500	
MM16223	Elachista atricomella	658	M	J. Tabell	ex larva em. 11-Jun-2008	Estonia	Vasalemma	59,24	24,29		Dactylis glomerata 19.V.2008
MM17256	Elachista atricomella	591		Aalto, Raesaenen	06-Jun-2006	Sweden	Oland, Graeborg	56,66	16,59		
MM17257	Elachista atricomella	658		Aalto, Raesaenen	06-Jun-2006	Sweden	Oland, Graeborg	56,66	16,59		
MM18546	Elachista atricomella	658		Ingvar Svensson	23-Jul-2008	Sweden	Gotland, Totta Blaehaell	57,53	18,11		
MM18547	Elachista atricomella	658		Ingvar Svensson	19-Jul-1999	Sweden	Gotland, Norrlanda	57,50	18,60		
MM19862	Elachista atricomella	658		Ingvar Svensson	11-Jul-2008	Sweden	Uppland, Haeveroe, Vaesternaes	59,70	18,70		
MM20022	Elachista atricomella	658	M	J. Tabell	08-Jun-2008	Estonia	Saaremaa, Vasalemma	59,24	24,28		
MM20023	Elachista atricomella	658	F	J. Tabell	15-Jun-2008	Estonia	Saaremaa, Vasalemma	59,24	24,28		
MM20918	Elachista atricomella	658	M	J. Junnilainen	09-May-2000	Estonia	Tallinn	59,40	24,70		
MM20937	Elachista atricomella	658	M	J. Junnilainen	21-Jun-2001	Bulgaria	Pirin, Popovi Livadi	41,53	23,61	1300	

MM20938	Elachista atricomella	658	M	J. Junnilainen	21-Jun-2001	Bulgaria	Pirin, Popovi Livadi	41,53	23,61	1300	
MM20940	Elachista atricomella	658	M	J-P. Kaitila	31-May-2002	Bulgaria	Kresna	41,72	23,16		
MM20942	Elachista atricomella	658	M	J-P. Kaitila	18-May-2003	Greece	Leptokaria 15 km W, Olympos	40,10	22,30	750	
MM20987	Elachista atricomella	658	M	J. Junnilainen	01-Jun-2008	Croatia	Platak	45,41	14,56	1000	
MM20988	Elachista atricomella	658	M	J. Junnilainen	01-Jun-2008	Croatia	Platak	45,41	14,56	1000	
MM20989	Elachista atricomella	658	M	J. Junnilainen	01-Jun-2008	Croatia	Platak	45,41	14,56	1000	
MM21386	Elachista atricomella	658	M	I. Richter	30-May-2003	Slovakia	Vysehrad	48,80	18,60		
MM21387	Elachista atricomella	658	M	Z. Tokar	03-Jun-2005	Slovenia	Pterijnski kras	45,57	13,91		
MM21444	Elachista atricomella	658	M	O. Karsholt	17-May-1994	Greece	Macedonia, Olympos mts.	40,05	22,25		
MM21445	Elachista atricomella	532	M	O. Karsholt	21-May-1994	Greece	Macedonia, Olympos mts.	40,05	22,25		
MM21447	Elachista atricomella	658	M	O. Karsholt	28-Aug-1991	Austria	Tirol, Kals	47,00	12,64	1100	
MM17656	Elachista baikalica	658		K. Nupponen, J. Kaitila, J. Junnilainen, M. Ahola	28-Jun-1996	Russia	Chelyabinsk, South Ural, Miass town, Ilmen State Reserve	55,01	60,10	350	
MM16162	Elachista bifasciella	658	M	J.Tabell	16-Jun-1905	Sweden	Skane, Roessjoeholm	56,20	13,20		L. Kaila prep. 1788
MM16163	Elachista bifasciella	627	F	J.Tabell	16-Jun-1905	Sweden	Skane, Roessjoeholm	56,20	13,20		
MM16164	Elachista bifasciella	658	F	J.Tabell	16-Jun-1905	Sweden	Skane, Roessjoeholm	56,20	13,20		
MM16165	Elachista bifasciella	658	F	J.Tabell	16-Jun-1905	Sweden	Skane, Roessjoeholm	56,20	13,20		
MM16167	Elachista bifasciella	658	M	P. Huemer	24-Jul-1995	Italy	South Tyrol, Ritten, Obergruenwald	46,55	11,42		
MM18548	Elachista bifasciella	658		Ingvar Svensson	ex larva 16.-18.5.2009	Sweden	Sk, Everoed	55,90	14,08		
MM18549	Elachista bifasciella	658		Ingvar Svensson	ex larva 16.-18.5.2009	Sweden	Sk, Everoed	55,90	14,08		
MM21664	Elachista bifasciella	658	F	L. Kaila, J. Tabell	26-May-2011	Switzerland	Linthal 3 km SW	46,88	8,96		
MM21665	Elachista bifasciella	658	F	L. Kaila, J. Tabell	24-May-2011	Switzerland	Linthal 3 km SW	46,88	8,96		
MM02669	Elachista canapennella	658		Marko Mutanen, Panu Vaelimaeki		Finland	Karelia australis, Imatra	61,11	28,80		
MM02670	Elachista canapennella	658		Marko Mutanen, Panu Vaelimaeki		Finland	Karelia australis, Imatra	61,11	28,80		
MM05360	Elachista canapennella	658		Marko Mutanen	-24.5.2006	Finland	Regio Aboensis, Turku	60,44	22,20		
MM05361	Elachista canapennella	637		Marko Mutanen	-24.5.2006	Finland	Regio Aboensis, Turku	60,44	22,20		
MM06187	Elachista canapennella	658		Marko Mutanen, Tomi Mutanen	18.-17.6.2007	Finland	Karelia ladogensis, Parikkala	61,64	29,64		
MM06302	Elachista canapennella	658	M	Marko Mutanen, Nestori Mutanen, Anttoni Mutanen	27-Jun-2007	Finland	Lapponia kemensis pars orientalis, Sodankylä	68,22	27,22		
MM06523	Elachista canapennella	658	F	Marko Mutanen, Nestori Mutanen	03-Jul-2007	Finland	Ostrobottnia borealis pars australis, Hailuoto	65,05	24,88		
MM06565	Elachista canapennella	658	M	Marko Mutanen, Anttoni Mutanen, Nestori Mutanen	03-Jul-2007	Finland	Ostrobottnia borealis pars australis, Hailuoto	65,00	24,69		

MM09868	Elachista canapennella	658		Marko Mutanen	17-Jun-2008	Finland	Karelia australis, Joutseno	61,06	28,72		
MM13896	Elachista canapennella	658		Marko Mutanen	18-May-2009	Finland	Regio Aboensis, Turku	60,33	22,24		
MM13967	Elachista canapennella	658		Marko Mutanen	05-Jun-2009	Finland	Ostrobottnia borealis pars australis, Kiiminki	65,07	23,81		
MM14269	Elachista canapennella	658		family Marko Mutanen	30-Jun-2009	Finland	Aland Islands, Geta	60,33	19,91		
MM14445	Elachista canapennella	658		Marko Mutanen, Nestori Mutanen, Anttoni Mutanen	09-Jul-2009	Finland	Ostrobottnia kajanensis, Ristijärvi	64,43	28,18		
MM14542	Elachista canapennella	658		Marko Mutanen	10-Jul-2009	Finland	Ostrobottnia borealis pars australis, Hailuoto	64,94	24,72		
MM16150	Elachista canapennella	658	M	J. Jalava, J. Kullberg	02-Jun-1995	Russia	Krasnoyarsk Krai, Krasnojarsk kr., Tanzybei	53,13	92,88		L. Kaila prep. 1838
MM16151	Elachista canapennella	658	M	J. Jalava, J. Kullberg	03-Jun-1995	Russia	Krasnoyarsk Krai, Krasnojarsk kr., Tanzybei	53,13	92,88		L. Kaila prep. 1839
MM16153	Elachista canapennella	658	M	J. Jalava, J. Kullberg	05-Jun-1995	Russia	Krasnoyarsk Krai, Krasnojarsk kr., Tanzybei	53,13	92,88		
MM18826	Elachista canapennella	658		Marko Mutanen	28.-31.7.2003	Finland	Nylandia, Kirkkonummi	59,93	24,32		
MM20905	Elachista canapennella	658	M	K. Nupponen	11-Jul-2003	Russia	Sverdlovskaya, Sverdlovsk district, North Ural, near Kytlym village, Kosvinskij Kamen	59,52	59,11	600	L. Kaila Prep. 4079
MM21092	Elachista canapennella	658		Tomi Mutanen	7.-8.8.2011	Finland	Regio aboensis, Salo	60,34	23,11		
MM10317	Elachista compsa	658		Tomi Mutanen	02-Jul-2008	Finland	Regio kuusamoensis, Kuusamo	66,31	29,45		
MM10423	Elachista compsa	658		Marko Mutanen	27-Jun-2005	Finland	Ostrobottnia borealis pars australis, Kiiminki	65,07	25,73		
MM10424	Elachista compsa	658		Marko Mutanen	27-Jun-2005	Finland	Ostrobottnia borealis pars australis, Kiiminki	65,07	25,73		
MM12148	Elachista compsa	658		Marko Mutanen, Panu Vaelimaeki	2007	Finland	Nylandia, Hanko	59,84	23,24		
MM12149	Elachista compsa	658		Marko Mutanen, Panu Vaelimaeki	2007	Finland	Nylandia, Hanko	59,84	23,24		
MM18827	Elachista compsa	658		Marko Mutanen	17-Jun-2008	Finland	Savonia australis, Joutseno	61,06	28,72		
MM20043	Elachista compsa	658	M	J. Tabell	larva 2009, em. 2010	Finland	Tavastia australis, Hartola	61,59	25,94		
MM04316	Elachista deriventa	621		Marko Mutanen, Panu Vaelimaeki	18-Jun-2005	Finland	Tavastia australis, Loppi	60,67	24,22		
MM14008	Elachista deriventa	658		Marko Mutanen	13.-14.6.2009	Finland	Karelia borealis, Tuupovaara	62,38	30,91		
MM14009	Elachista deriventa	658		Marko Mutanen	13.-14.6.2009	Finland	Karelia borealis, Tuupovaara	62,38	30,91		
MM14010	Elachista deriventa	658		Marko Mutanen	13.-14.6.2009	Finland	Karelia borealis, Tuupovaara	62,38	30,91		
MM14011	Elachista deriventa	658		Marko Mutanen	13.-14.6.2009	Finland	Karelia borealis, Tuupovaara	62,38	30,91		
MM14012	Elachista deriventa	658		Marko Mutanen	13.-14.6.2009	Finland	Karelia borealis, Tuupovaara	62,38	30,91		

MM15197	Elachista deriventa	658		Ali Karhu	11-Sep-2010	Finland	Karelia borealis, Liperi	62,49	29,13	larca on Calamagrostis sp., no voucher
MM16310	Elachista deriventa	658	M	J. Tabell	17-Jun-2008	Finland	Tavastia australis, Hartola	61,43	25,91	GP 4652 JT
MM05677	Elachista diederichsiella	658	M	Petri Hirvonen, Panu Vaelimaeki	03-Jul-2007	Finland	Lapland, Lapponia inarensis, Utsjoki	69,40	25,83	
MM05678	Elachista diederichsiella	658	F	Petri Hirvonen, Panu Vaelimaeki	03-Jul-2007	Finland	Lapland, Lapponia inarensis, Utsjoki	69,52	25,87	
MM06224	Elachista diederichsiella	658		Marko Mutanen	19-Jun-2007	Finland	Karelia australis, Joutseno	61,06	28,72	
MM08330	Elachista diederichsiella	658		Marko Mutanen	5.-12.7.2006	Finland	Ostrobottnia borealis pars borealis, Tornio	65,90	24,45	
MM09219	Elachista diederichsiella	658		Petri Hirvonen, Marko Mutanen	May 2009	Finland	Ostrobottnia borealis pars borealis, Tornio	65,79	24,23	
MM09240	Elachista diederichsiella	658	M	Petri Hirvonen, Marko Mutanen	ex larva 2009	Finland	Ostrobottnia borealis pars borealis, Tornio	65,79	24,23	
MM09241	Elachista diederichsiella	658	M	Petri Hirvonen, Marko Mutanen	ex larva 2009	Finland	Ostrobottnia borealis pars borealis, Tornio	65,79	24,23	
MM15491	Elachista diederichsiella	657[1n]	M	Jalava, Kullberg, Koponen	12-Jul-1994	Russia	Polar Ural, Krasnyi Kamen	66,08	65,17	L. Kaila prep. 1372
MM16191	Elachista diederichsiella	658	M	A. Albrecht	04-Jun-1990	Estonia	Paernu r., Nigula, Salupeaksi	58,30	24,40	L. Kaila prep. 2711
MM16192	Elachista diederichsiella	658	M	A. Albrecht	04-Jun-1990	Estonia	Paernu r., Nigula, Salupeaksi	58,30	24,40	L. Kaila prep. 451
MM16193	Elachista diederichsiella	658	M	K. Mikkola	13-Jul-2003	Norway	Fn, Berlevaeg, Guldofjord	70,80	29,00	L. Kaila prep. 3730
MM16194	Elachista diederichsiella	658	M	K. Mikkola	13-Jul-2003	Norway	Fn, Berlevaeg, Guldofjord	70,80	29,00	
MM16195	Elachista diederichsiella	658	M	K. Mikkola	13-Jul-2003	Norway	Fn, Berlevaeg, Guldofjord	70,80	29,00	
MM16197	Elachista diederichsiella	658	M	Jalava, Kullberg, Koponen	08-Jul-1994	Russia	Polar Ural, Krasnyi Kamen	66,08	65,17	L. Kaila prep. 1958
MM16198	Elachista diederichsiella	658	M	Jalava, Kullberg, Koponen	09-Jul-1994	Russia	Polar Ural, Krasnyi Kamen	66,08	65,17	L. Kaila prep. 1370
MM16199	Elachista diederichsiella	658	M	Jalava, Kullberg, Koponen	10-Jul-1994	Russia	Polar Ural, Krasnyi Kamen	66,08	65,17	L. Kaila prep. 1957
MM16200	Elachista diederichsiella	633	M	Jalava, Kullberg, Koponen	11-Jul-1994	Russia	Polar Ural, Krasnyi Kamen	66,08	65,17	L. Kaila prep. 1956
MM16315	Elachista diederichsiella	658	F	R. Siloaho	01-Jul-2008	Finland	Lapland, Lapponia inarensis, Utsjoki	69,95	27,29	
MM16316	Elachista diederichsiella	658	F	J. Tabell	ex larva 31.5.2007	Finland	Karelia australis, Joutseno	61,07	28,70	Milium effusum 11.V.2007
MM17965	Elachista diederichsiella	658		Marko Mutanen, Nestori Mutanen, Anttoni Mutanen	11-Jul-2010	Finland	Lapponia inarensis, Utsjoki	69,43	26,01	
MM17966	Elachista diederichsiella	658		Marko Mutanen, Nestori Mutanen, Anttoni Mutanen	11-Jul-2010	Finland	Lapponia inarensis, Utsjoki	69,43	26,01	
MM17967	Elachista diederichsiella	658		Marko Mutanen, Nestori Mutanen, Anttoni Mutanen	11-Jul-2010	Finland	Lapponia inarensis, Utsjoki	69,43	26,01	

MM17968	Elachista diederichsiella	658		Marko Mutanen, Nestori Mutanen, Anttoni Mutanen	11-Jul-2010	Finland	Lapponia inarensis, Utsjoki	69,43	26,01		
MM17969	Elachista diederichsiella	658		Marko Mutanen, Nestori Mutanen, Anttoni Mutanen	11-Jul-2010	Finland	Lapponia inarensis, Utsjoki	69,43	26,01		
MM17970	Elachista diederichsiella	658		Marko Mutanen, Nestori Mutanen, Anttoni Mutanen	11-Jul-2010	Finland	Lapponia inarensis, Utsjoki	69,43	26,01		
MM20881	Elachista diederichsiella	658	F	T. Nupponen, K. Nupponen	08-Jul-2000	Russia	Altai Krai, Altai Mts., Sarlyk region	51,00	85,58	1450	
MM20890	Elachista diederichsiella	657[1n]	M	T. Nupponen, K. Nupponen	05-Jul-2000	Russia	Altai Krai, Altai Mts., Chuja valley 5 km SE Aktash village	50,25	87,67	1500	
MM20903	Elachista diederichsiella	658	F	K. Nupponen	11-Jul-2003	Russia	Sverdlovskaya, Sverdlovsk district, North Ural, near Kytlym village, Kosvinskij Kamen	59,52	59,11	600	
MM20925	Elachista diederichsiella	658	F	Timo Nupponen, Kari Nupponen	14-Jul-1999	Russia	Tyumenskaya, Tjumen district, Polar-Ural, Labytnangi	66,66	65,66	50	
MM20936	Elachista diederichsiella	658	M	K. Nupponen	17-Jun-2002	Russia	Irkutsk, Irkutsk prov., Sludjanka village 2 km SE, Lake Baikal shope slope	51,67	103,7 2	500	
MM20976	Elachista diederichsiella	658	M	Timo Nupponen, Kari Nupponen	10-Jul-1999	Russia	Tyumenskaya, Tjumen district, Polar-Ural, Krasnyi Kamen	66,93	65,66	200	
MM21424	Elachista diederichsiella	658	M	G. Stahls	11-Jun-1990	Russia	Altai Krai, Gorno-Altaisk, Artybash	51,70	87,20		
MM16159	Elachista dimicatella	425	M	Kullberg, Lievonen	3.-5.6.2003	Ukraine	Ivano-Frankovsk, Burkyt, Verchovna distr.	47,90	24,73		
MM16160	Elachista dimicatella	658	M	Kullberg, Lievonen	24.-26.6.2003	Ukraine	Ivano-Frankovsk, Mt. Chivchen, Verchovna distr.	47,86	24,71		L. Kaila prep. 4827
MM16161	Elachista dimicatella	658	M	Kullberg, Lievonen	24.-26.6.2003	Ukraine	Ivano-Frankovsk, Mt. Chivchen, Verchovna distr.	47,86	24,71		L. Kaila prep. 4853
MM10509	Elachista elegans	658	M	Esko Saarela	ex larva 2009	Finland	Tavastia australis, Tampere	61,54	23,80		mining early, dark forest
MM10510	Elachista elegans	658	F	Esko Saarela		Finland	Tavastia australis, Tampere	61,54	23,80		ex larva 2009, mining early, dark forest
MM10511	Elachista elegans	658	F	Esko Saarela	ex larva 2009	Finland	Tavastia australis, Tampere	61,54	23,80		mining early, dark forest
MM10512	Elachista elegans	658	M	Esko Saarela	14-Jul-2009	Finland	Tavastia australis, Pälkäne	61,31	26,07		late flight time, light forest
MM10514	Elachista elegans	658	M	Leo Sippola	15-Jul-2009	Finland	Tavastia australis, Pälkäne	61,31	26,07		late flight time, light forest
MM10515	Elachista elegans	658	M	Leo Sippola	18-Jul-2009	Finland	Tavastia australis, Pälkäne	61,31	26,07		late flight time, light forest

MM21158	Elachista elegans	658		Marko Mutanen, Anttoni Mutanen, Nestori Mutanen	29.-30.7.2011	Finland	Savonia australis, Imatra	61,16	28,75	
MM23191	Elachista elegans	658		Marko Mutanen	27-Jul-2005	Finland	Regio aboensis, Dragsfjärd, Taalintehdas	60,02	22,43	
MM08409	Elachista eskoi	658		Marko Mutanen	2003	Finland	Ostrobottnia borealis pars australis, Kiiminki	65,07	25,73	
MM08410	Elachista eskoi	658		Marko Mutanen	2003	Finland	Ostrobottnia borealis pars australis, Kiiminki	65,07	25,73	
MM10428	Elachista eskoi	658		Marko Mutanen	30-Jun-2005	Finland	Ostrobottnia borealis pars australis, Oulu	64,98	25,31	
MM10429	Elachista eskoi	658		Marko Mutanen	30-Jun-2005	Finland	Ostrobottnia borealis pars australis, Oulu	64,98	25,31	
MM10430	Elachista eskoi	658		Marko Mutanen	3.-4.7.2003	Finland	Regio kuusamoensis, Kuusamo	66,32	29,23	
MM14159	Elachista eskoi	658		Marko Mutanen	23-Jun-2009	Finland	Ostrobottnia borealis pars australis, Oulu	64,99	25,41	
MM14160	Elachista eskoi	658		Marko Mutanen	23-Jun-2009	Finland	Ostrobottnia borealis pars australis, Oulu	64,99	25,41	
MM20025	Elachista eskoi	658	M	J. Tabell	16-Jun-2007	Finland	Kymenlaakso, Karelia australis, Virolahti	60,52	27,70	
MM20026	Elachista eskoi	658	M	J. Tabell	17-Jun-2007	Finland	Kymenlaakso, Karelia australis, Virolahti	60,52	27,70	
MM20027	Elachista eskoi	658	M	J. Tabell	17-Jun-2007	Finland	Kymenlaakso, Karelia australis, Virolahti	60,52	27,70	
MM21208	Elachista eskoi	658		Matti Ahola	23-Jun-2009	Finland	Ostrobottnia media, Reisjärvi	63,59	23,02	
MM21216	Elachista eskoi	658	M	Marko Mutanen, Anttoni Mutanen, Nestori Mutanen	23-Jun-2011	Finland	Ostrobottnia borealis pars australis, Kiiminki	65,12	25,83	
MM21253	Elachista eskoi	658		Marko Mutanen	30-Jun-2005	Finland	Ostrobottnia borealis pars australis, Oulu	65,02	25,41	
MM00685	Elachista excelsicola	612		Tomi Mutanen	28-Jun-2006	Finland	Obb, Ranua	65,88	26,34	
MM03441	Elachista excelsicola	658		Marko Mutanen	6.-8.7.2006	Finland	Lapland, Lapponia enontekiensis, Enontekiö	69,00	20,74	
MM03983	Elachista excelsicola	670		Marko Mutanen	29-Jun-2006	Finland	Oba, Kiiminki	65,07	25,73	gen.studied, identification not confirmed
MM04313	Elachista excelsicola	612		Petri Hirvonen	02-Jul-2006	Finland	Le, Enontekiö	69,00	20,74	
MM05881	Elachista excelsicola	658	F	Erkki M. Laasonen, Leena Laasonen	05-Jul-2007	Finland	Lapponia inarensis, Inari	68,43	27,39	
MM05884	Elachista excelsicola	612	M	Erkki M. Laasonen, Leena Laasonen	03-Jul-2007	Finland	Li, Inari	68,72	27,69	
MM05885	Elachista excelsicola	658	M	Erkki M. Laasonen, Leena Laasonen	03-Jul-2007	Finland	Lapponia inarensis, Inari	68,72	27,69	
MM05886	Elachista excelsicola	658	F	Erkki M. Laasonen, Leena Laasonen	03-Jul-2007	Finland	Lapponia inarensis, Inari	68,72	27,69	
MM05936	Elachista excelsicola	658	M	Erkki M. Laasonen, Leena Laasonen	14-Jul-2008	Finland	Lapponia inarensis, Inari	68,44	27,39	
MM05938	Elachista excelsicola	658	M	Erkki M. Laasonen, Leena Laasonen	14-Jul-2008	Finland	Lapponia inarensis, Inari	68,44	27,39	

MM08590	Elachista excelsicola	658	M	Marko Mutanen	23-Jun-2008	Finland	Ostrobottnia borealis pars australis, Kiiminki	65,07	25,73		
MM10426	Elachista excelsicola	658		Marko Mutanen	21-Jun-2005	Finland	Ostrobottnia borealis pars australis, Kiiminki	65,07	25,73		
MM15562	Elachista excelsicola	658	M	Marko Mutanen	08-Jul-1997	Finland	Regio kuusamoensis, Kuusamo	66,32	29,23		
MM15564	Elachista excelsicola	571	M	Tomi Mutanen	21-Jun-1995	Finland	Ostrobottnia borealis pars borealis, Tornio	65,86	24,37		
MM16219	Elachista excelsicola	658	M	R. Siloaho	30-Jun-2009	Finland	Lapponia inarensis, Saariselkä	68,39	27,24		GP 4458 JT
MM17655	Elachista excelsicola	658		Jari Kaitila	20-Jul-2000	Finland	Lapponia inarensis, Utsjoki	69,74	27,00		
MM17971	Elachista excelsicola	658		Marko Mutanen, Nestori Mutanen, Anttoni Mutanen	year 2010	Finland	Ostrobottnia borealis pars australis, Kiiminki	65,07	25,72		
MM18589	Elachista excelsicola	658		Bengt A. Bengtsson	03-Jul-2004	Sweden	To, Abisko, Nissunsnakk	68,25	18,90		
MM19855	Elachista excelsicola	658		T. Mutanen	03-Jul-1995	Finland	Lapland, Lapponia kemensis pars orientalis, Pelkosenniemi	67,14	27,69		
MM19893	Elachista excelsicola	658	F	Erkki Laasonen, Leena Laasonen		Finland	Lapponia inarensis, Inari	69,80	26,29		
MM20907	Elachista excelsicola	658	M	Jalava, Tammaru	10-Jul-1997	Russia	Magadan, Magadanskaya Oblast, nr. Gulu village, biological station, Kontakt	61,85	147,6 7	850	
MM20922	Elachista excelsicola	658	M	Timo Nupponen, Kari Nupponen	12-Jul-1999	Russia	Tyumenskaya, Tjumen district, Polar-Ural, Krasnyi Kamen	66,93	65,66	200	
MM20923	Elachista excelsicola	658	M	Timo Nupponen, Kari Nupponen	12-Jul-1999	Russia	Tyumenskaya, Tjumen district, Polar-Ural, Krasnyi Kamen	66,93	65,66	200	
MM20924	Elachista excelsicola	658	M	Timo Nupponen, Kari Nupponen	10-Jul-1999	Russia	Tyumenskaya, Tjumen district, Polar-Ural, Krasnyi Kamen	66,93	65,66	200	
MM21183	Elachista excelsicola	658		Marko Mutanen, Anttoni Mutanen, Nestori Mutanen	28-Jun-2011	Finland	Ostrobottnia kajanensis, Kuhmo	63,91	29,84		
MM21202	Elachista excelsicola	658		Marko Mutanen	27-Jun-2011	Finland	Regio kuusamoensis, Kuusamo	66,36	29,60		
MM22226	Elachista excelsicola	633	M	Jalava, Kullberg, Koponen	02-Jul-1994	Russia	Polar Ural, Krasnyi Kamen	66,55	65,10	400	
MM22607	Elachista excelsicola	658	M	J.-P. Kaitila	12-Jul-2008	Finland	Lapponia enontekiensis, Enontekiö, Toshkaljoki	69,16	21,58		
MM22608	Elachista excelsicola	658	M	J.-P. Kaitila	11-Jul-2008	Finland	Lapponia enontekiensis, Enontekiö, Toshkaljoki	69,16	21,58		
MM16175	Elachista fuscofrontella	658	M	A. Albrecht	07-Jun-1990	Estonia	Tartu, Jaervselja, Saki	58,31	27,29		
MM21451	Elachista fuscofrontella	658	M	K. Nupponen	09-May-2005	Russia	Orenburg, South Ural, Pokrovka village 20 km S, Schibendy valley	50,68	54,45	200	
MM20939	Elachista griseella	658	M	J-P. Kaitila	27-Apr-2002	Cyprus	Kypros, Pano Panagia	34,91	32,63		L. Kaila Prep. 3991

MM16138	Elachista herrichii	658	M	K. Nupponen	20-May-2004	Russia	Orenburg, South Ural, Guberlinsky Mts., Orsk 40 km W, near Guberlja village	51,25	58,08	340	L. Kaila prep. 4846
MM16139	Elachista herrichii	658	M	K. Nupponen	20-May-2004	Russia	Orenburg, South Ural, Guberlinsky Mts., Orsk 40 km W, near Guberlja village	51,25	58,08	340	L. Kaila prep. 4394
MM16141	Elachista herrichii	658	M	Timo Nupponen, Kari Nupponen	28-May-1998	Russia	Chelyabinsk, South Ural, Mednogorsk 20 km S, near Kidriasovo village	51,21	57,61	350	L. Kaila prep. 3334
MM16142	Elachista herrichii	658	M	K. Nupponen	11-May-2005	Russia	Orenburg, South Ural, Pokrovka village 20 km S, Schibendy valley	50,68	54,45	200	L. Kaila prep. 4838
MM16143	Elachista herrichii	658	M	Yu. Budashkin	24-Jul-1996	Ukraine	Krym, Karadagh	44,90	35,20		
MM00863	Elachista humilis	658		Marko Mutanen	20.-21.6.2006	Finland	Aland Islands, Kökar	59,94	20,90		
MM02663	Elachista humilis	658		Marko Mutanen, Panu Vaelimaeki		Finland	Karelia australis, Imatra	61,11	28,80		
MM06168	Elachista humilis	658		Marko Mutanen, Tomi Mutanen	18.-17.6.2007	Finland	Karelia ladogensis, Parikkala	61,64	29,64		
MM06171	Elachista humilis	658		Marko Mutanen, Tomi Mutanen	18.-17.6.2007	Finland	Karelia ladogensis, Parikkala	61,64	29,64		
MM06222	Elachista humilis	658		Marko Mutanen	19-Jun-2007	Finland	Karelia australis, Joutseno	61,06	28,72		
MM08064	Elachista humilis	658		Marko Mutanen, Panu Vaelimaeki	3.-17.7.2006	Finland	Lapponia kemensis pars occidentalis, Kolari	67,28	23,75		
MM08065	Elachista humilis	658		Marko Mutanen, Panu Vaelimaeki	3.-17.7.2006	Finland	Lapponia kemensis pars occidentalis, Kolari	67,28	23,75		
MM08660	Elachista humilis	658		Marko Mutanen	19-Jul-2008	Finland	Aland Islands, Lemland	60,01	20,13		
MM09744	Elachista humilis	649		Marko Mutanen, Meelika Mutanen, Atsalea Mutanen, Sylvia Mutanen	22-Jul-2008	Finland	Aland Islands, Eckerö	60,28	19,58		
MM09867	Elachista humilis	658		Marko Mutanen	17-Jun-2008	Finland	Karelia australis, Joutseno	61,06	28,72		
MM14237	Elachista humilis	658		Marko Mutanen, Nestori Mutanen, Anttoni Mutanen	29-Jun-2009	Finland	Aland Islands, Eckerö	60,24	19,59		
MM14238	Elachista humilis	658		Marko Mutanen, Nestori Mutanen, Anttoni Mutanen	29-Jun-2009	Finland	Aland Islands, Eckerö	60,24	19,59		
MM14460	Elachista humilis	658		Marko Mutanen, Nestori Mutanen, Anttoni Mutanen	09-Jul-2009	Finland	Ostrobottnia kajanensis, Ristijärvi	64,39	28,20		
MM15298	Elachista humilis	658	M	A. Albrecht	31-May-1990	Estonia	Saaremaa, Loopollu	58,40	41416 ,00		
MM15560	Elachista humilis	642	F	Marko Mutanen	11.-12.7.2000	Finland	Ostrobottnia borealis pars australis, Pudasjärvi	65,70	27,65		
MM16117	Elachista humilis	658	M	E. Laasonen	13.-15.7.2002	Sweden	Skane, Lund	55,70	13,10		
MM16118	Elachista humilis	619	M	E. Laasonen	07-Jul-2002	Sweden	Skane, Lund	55,70	13,10		

MM17392	Elachista humilis	658		Juhani Itaemies	22-Jun-2003	Finland	Satakunda, Eurajoki	61,19	21,42		Deschampsia caespitosa
MM17942	Elachista humilis	658		Sami Haapala	22-Jun-2009	Finland	Savonia australis, Lappeenranta	61,06	28,72		
MM17943	Elachista humilis	658		Sami Haapala	23-Jun-2009	Finland	Karelia lagodensis, Parikkala	61,55	29,57		
MM20239	Elachista humilis	658	M	J. Tabell	17-Jul-2011	Austria	Niederalpi	47,68	15,37		
MM20921	Elachista humilis	658	M	K. Nupponen, J. Junnilainen	23-Jul-1997	Poland	Tatra mnts., Bobrowiec, Mnichy Chocholowskie	49,24	19,79	1450	
MM20928	Elachista humilis	655[3n]	M	K. Nupponen, J. Junnilainen	23-Jul-1997	Poland	Tatra mnts., Bobrowiec, Mnichy Chocholowskie	49,24	19,79	1450	
MM22597	Elachista humilis	658	M	J. Kullberg., T. Lievonen	25-Aug-2003	Ukraine	Ivano-Frankovsk, Verkhovyna, Mt. Chiven	47,86	24,71	1675	
MM16147	Elachista irenae	658	M	K. Nupponen, J. Junnilainen	28-Jul-1997	Poland	Tatra Mts, Kominiarski Wierch	49,24	19,83		
MM16149	Elachista irenae	658	M	K. Nupponen, J. Junnilainen	30-Jul-1997	Poland	Tatra Mts, Kominiarski Wierch	49,24	19,83		
MM00687	Elachista kilmunella	658		Tomi Mutanen	28-Jun-2006	Finland	Lapland, Ostrobottnia borealis pars borealis, Ranua	65,88	26,34		
MM03901	Elachista kilmunella	658		Marko Mutanen	27-Jun-2006	Finland	Ostrobottnia borealis pars australis, Pudasjärvi	65,39	26,91		
MM03902	Elachista kilmunella	658		Marko Mutanen	27-Jun-2006	Finland	Ostrobottnia borealis pars australis, Pudasjärvi	65,39	26,91		
MM04003	Elachista kilmunella	658		Marko Mutanen	13.-14.6.2006	Finland	North Karelia, Karelia borealis, Kesälahti	61,79	29,71		
MM05876	Elachista kilmunella	658	M	Erkki M. Laasonen, Leena Laasonen	05-Jul-2007	Finland	Lapponia inarensis, Inari	68,43	27,39		
MM05877	Elachista kilmunella	658	F	Erkki M. Laasonen, Leena Laasonen	05-Jul-2007	Finland	Lapponia inarensis, Inari	68,43	27,39		
MM05878	Elachista kilmunella	637	M	Erkki M. Laasonen, Leena Laasonen	05-Jul-2007	Finland	Li, Inari	68,43	27,39		
MM05879	Elachista kilmunella	613	F	Erkki M. Laasonen, Leena Laasonen	05-Jul-2007	Finland	Li, Inari	68,43	27,39		
MM05880	Elachista kilmunella	658	M	Erkki M. Laasonen, Leena Laasonen	05-Jul-2007	Finland	Lapponia inarensis, Inari	68,43	27,39		
MM05882	Elachista kilmunella	670	M	Erkki M. Laasonen, Leena Laasonen	03-Jul-2007	Finland	Li, Inari	68,72	27,69		
MM05928	Elachista kilmunella	658	M	Erkki M. Laasonen, Leena Laasonen	03-Jul-2008	Finland	Ostrobottnia borealis pars borealis, Keminmaa	65,89	24,63		
MM05929	Elachista kilmunella	658	M	Erkki M. Laasonen, Leena Laasonen	03-Jul-2008	Finland	Ostrobottnia borealis pars borealis, Keminmaa	65,89	24,63		
MM05930	Elachista kilmunella	658	M	Erkki M. Laasonen, Leena Laasonen	03-Jul-2008	Finland	Ostrobottnia borealis pars borealis, Keminmaa	65,89	24,63		
MM05931	Elachista kilmunella	658	M	Erkki M. Laasonen, Leena Laasonen	14-Jul-2008	Finland	Lapponia inarensis, Inari	68,44	27,39		
MM05932	Elachista kilmunella	658	M	Erkki M. Laasonen, Leena Laasonen	14-Jul-2008	Finland	Lapponia inarensis, Inari	68,44	27,39		
MM05935	Elachista kilmunella	658	M	Erkki M. Laasonen, Leena Laasonen	14-Jul-2008	Finland	Lapponia inarensis, Inari	68,44	27,39		

MM05937	Elachista kilmunella	632	M	Erkki M. Laasonen, Leena Laasonen	14-Jul-2008	Finland	Lapponia inarensis, Inari	68,44	27,39		
MM06594	Elachista kilmunella	658	M	Tomi Mutanen, Marko Mutanen, Nestori Mutanen, Anttoni Mutanen	6.-7.7.2007	Finland	Karelia borealis, Ilomantsi	62,51	30,88		
MM09242	Elachista kilmunella	658	M	Petri Hirvonen	07-Jul-2009	Finland	Regio kuusamoensis, Kuusamo	66,14	23,90		
MM09251	Elachista kilmunella	658	M	Petri Hirvonen	07-Jul-2009	Finland	Regio kuusamoensis, Kuusamo	66,14	23,90		
MM15563	Elachista kilmunella	658	M	Marko Mutanen	24-Jun-2004	Finland	Ostrobottnia borealis pars australis, Kiiminki	65,07	25,73		
MM16190	Elachista kilmunella	658	M	Jalava, Kullberg, Koponen	08-Jul-1994	Russia	Polar Ural, Krasnyi Kamen	66,08	65,17		L. Kaila prep. 2091
MM16207	Elachista kilmunella	658	M	J. Tabell	18-Jun-2006	Finland	Tavastia australis, Hartola	61,59	26,02		GP 4253 JT
MM16208	Elachista kilmunella	658	M	J. Tabell	30-Jun-2006	Finland	Tavastia australis, Hartola	61,59	26,02		GP 3907 JT
MM17963	Elachista kilmunella	658		family Marko Mutanen	30-Jun-2010	Finland	Ostrobottnia kajanensis, Kuhmo	63,91	29,83		
MM18579	Elachista kilmunella	658		Ingvar Svensson	01-Jul-2002	Sweden	Jaemtland, Storlidfjaellet	64,77	13,93		
MM18580	Elachista kilmunella	658		Ingvar Svensson	01-Jul-2002	Sweden	Jaemtland, Storlidfjaellet	64,77	13,93		
MM19892	Elachista kilmunella	658	M	Erkki Laasonen, Leena Laasonen		Finland	Lapland, Lapponia inarensis, Utsjoki	69,81	27,17		
MM20004	Elachista kilmunella	658	M	J. Tabell	17-Jun-2006	Finland	Tavastia australis, Hartola	61,60	26,03		GP 3908 JT
MM20005	Elachista kilmunella	658	M	J. Tabell	12-Jun-2009	Finland	Tavastia australis, Hartola	61,60	26,03		
MM21201	Elachista kilmunella	657[1n]		Marko Mutanen	27-Jun-2011	Finland	Regio kuusamoensis, Kuusamo	66,36	29,60		
MM21337	Elachista kilmunella	658	M	J. Junnilainen	01-Jul-2008	Finland	Kuusamo	66,40	29,40		
MM14194	Elachista krogeri	658		Panu Vaelimaeki	22-Jun-2009	Finland	Ostrobottnia borealis pars australis, Oulu	64,94	25,46		
MM14195	Elachista krogeri	658		Panu Vaelimaeki	22-Jun-2009	Finland	Ostrobottnia borealis pars australis, Oulu	64,94	25,46		
MM14196	Elachista krogeri	623		Panu Vaelimaeki	22-Jun-2009	Finland	Ostrobottnia borealis pars australis, Oulu	64,94	25,46		
MM14202	Elachista krogeri	658		MarkoMutanen	24-Jun-2009	Finland	Ostrobottnia borealis pars australis, Oulu	64,94	25,46		
MM14203	Elachista krogeri	658		MarkoMutanen	24-Jun-2009	Finland	Ostrobottnia borealis pars australis, Oulu	64,94	25,46		
MM14204	Elachista krogeri	658		MarkoMutanen	24-Jun-2009	Finland	Ostrobottnia borealis pars australis, Oulu	64,94	25,46		
MM16155	Elachista krogeri	658	M	Jalava, Kullberg, Koponen	12-Jul-1994	Russia	Ob Delta, Labytnangi	66,70	66,58		L. Kaila prep. 1368
MM16157	Elachista krogeri	658	M	Jalava, Kullberg, Koponen	12-Jul-1994	Russia	Ob Delta, Labytnangi	66,70	66,58		L. Kaila prep. 1205
MM16158	Elachista krogeri	658	M	Jalava, Kullberg, Koponen	07-Jul-1994	Russia	Ob Delta, Labytnangi	66,70	66,58		L. Kaila prep. 1206
MM20920	Elachista krogeri	654	M	Timo Nupponen, Kari Nupponen	14-Jul-1999	Russia	Tyumenskaya, Tjumen district, Polar-Ural, Labytnangi	66,66	65,66	50	L. Kaila Prep. 3631

MM20926	Elachista krogeri	595[1n]	M	Timo Nupponen, Kari Nupponen	14-Jul-1999	Russia	Tyumenskaya, Tjumen district, Polar-Ural, Labytnangi	66,66	65,66	50	
MM20927	Elachista krogeri	596[2n]	M	Timo Nupponen, Kari Nupponen	14-Jul-1999	Russia	Tyumenskaya, Tjumen district, Polar-Ural, Labytnangi	66,66	65,66	50	
BC ZSM Lep 53220	Elachista lastrella	658	F	Dr. Theo Gruenewald	22-May-2010	Germany	Bavaria, Oberpfalz, Regensburg, Nittendorf	49,03	11,96	400	
MM03980	Elachista leifi	658		Marko Mutanen	29-Jun-2006	Finland	Ostrobottnia borealis pars australis, Pudasjärvi	65,39	26,91		
MM05943	Elachista leifi	619	F	Erkki M. Laasonen, Leena Laasonen	14-Jul-2008	Finland	Lapponia inarensis, Inari	68,95	28,46		
MM10425	Elachista leifi	658		Marko Mutanen	30-Jun-2000	Finland	Ostrobottnia borealis pars australis, Pudasjärvi	65,70	27,65		
MM16249	Elachista leifi	658	M	J. Tabell	01-Jul-2009	Finland	Ostrobottnia borealis pars australis, Rovaniemi	66,45	25,66		GP 4355 JT
MM18150	Elachista leifi	658		Marko Mutanen	03-Jul-1995	Finland	Lapponia kemensis pars orientalis, Pelkosenniemi	67,19	27,88		Gen. prep. no. 533 M. Mutanen
MM06643	Elachista luticomella	658	M	Marko Mutanen	11-Jul-2007	Finland	Nylandia, Kirkkonummi	59,98	24,44		
MM06782	Elachista luticomella	658	M	Marko Mutanen	13-Jul-2007	Finland	Aland Islands, Maarianhamina	60,10	19,93		
MM06797	Elachista luticomella	658		Marko Mutanen	14-Jul-2007	Finland	Aland Islands, Sund	60,23	20,06		
MM08652	Elachista luticomella	658		Marko Mutanen	18-Jul-2008	Finland	Aland Islands, Hammarland	60,23	19,69		
MM09725	Elachista luticomella	658		Marko Mutanen, Meelika Mutanen, Atsalea Mutanen, Sylvia Mutanen	21-Jul-2008	Finland	Aland Islands, Geta	60,36	19,91		
MM20042	Elachista luticomella	658	M	J. Tabell	09-Jun-2008	Estonia	Saaremaa, Vasalemma	59,24	24,28		
MM00861	Elachista maculicerusella	658		Marko Mutanen	20.-21.6.2006	Finland	Aland Islands, Kökar	59,94	20,90		
MM02647	Elachista maculicerusella	658		Marko Mutanen, Panu Vaelimaeki	2006	Finland	Savonia australis, Imatra	61,11	28,80		genitalia examined
MM02648	Elachista maculicerusella	634		Marko Mutanen, Panu Vaelimaeki	2006	Finland	Savonia australis, Imatra	61,11	28,80		
MM03985	Elachista maculicerusella	658		Marko Mutanen	02-Aug-2006	Finland	Kymenlaakso, Karelia australis, Kotka	60,41	26,82		
MM03986	Elachista maculicerusella	658		Marko Mutanen	01-Aug-2006	Finland	Nylandia, Kirkkonummi	59,98	24,44		
MM09733	Elachista maculicerusella	658		Marko Mutanen, Meelika Mutanen, Atsalea Mutanen, Sylvia Mutanen	21-Jul-2008	Finland	Aland Islands, Geta	60,36	19,91		
MM12025	Elachista maculicerusella	658		Marko Mutanen, Panu Vaelimaeki	2007	Finland	Nylandia, Hanko	59,84	23,24		
MM14357	Elachista maculicerusella	658		Marko Mutanen	01-Jul-2009	Finland	Aland Islands, Sund	60,18	20,23		
MM15439	Elachista maculicerusella	658		B. Wikstrom	15.-16-May-2002	Russia	Moskovskaya, Moscow oblast, Stupino region	55,20	37,90		

MM15440	Elachista maculicerusella	658		B. Wikstrom	15.-16-May-2002	Russia	Moskovskaya, Moscow oblast, Stupino region	55,20	37,90		
MM15441	Elachista maculicerusella	658		B. Wikstrom	15.-16-May-2002	Russia	Moskovskaya, Moscow oblast, Stupino region	55,20	37,90		
MM15442	Elachista maculicerusella	658		A. Albrecht	03-Jun-1990	Estonia	Paernu r., Nigula	58,30	24,40		
MM15443	Elachista maculicerusella	658		A. Albrecht	03-Jun-1990	Estonia	Paernu r., Nigula	58,30	24,40		
MM15444	Elachista maculicerusella	658		M. Kozlov	25-Jul-1991	Russia	Murmansk, Murmansk, Luvenga, White sea cost	67,10	32,71		
MM15445	Elachista maculicerusella	658		M. Kozlov	25-Jul-1991	Russia	Murmansk, Murmansk, Luvenga, White sea cost	67,10	32,71		
MM15448	Elachista maculicerusella	658		L. Kaila	01-Jul-1990	Kazakhstan	Zailiskiy Alatau, Alma-Atinskij Nat.P.	43,08	77,25		
MM15449	Elachista maculicerusella	658		L. Kaila	01-Jul-1990	Kazakhstan	Zailiskiy Alatau, Alma-Atinskij Nat.P.	43,08	77,25		L. Kaila prep. 351
MM16230	Elachista maculicerusella	658	F	J. Tabell	ex larva em. 7-Jun-2008	Finland	Tavastia australis, Hartola	61,59	26,02		Calamagrostis epigejos 27.V.2008
MM16231	Elachista maculicerusella	658	M	P. Varalda	ex larva em. 3-Apr-2007	Italy	Piedmont, Morano sul Po	45,16	8,36		Phalaris arundinacea 19.III.2007
MM16232	Elachista maculicerusella	658	M	J. Tabell	07-Jun-2009	Finland	Tavastia australis, Sysmä	61,54	25,93		
MM16233	Elachista maculicerusella	658	F	P. Varalda	ex larva em. 3-Apr-2007,	Italy	Piedmont, Morano sul Po	45,16	8,36		Phalaris arundinacea 19.III.2007, GP 4251 JT
MM16980	Elachista maculicerusella	658	M	J. Junnilainen	02-Jun-2008	Croatia	Jartsebarsko	45,67	15,65		
MM17940	Elachista maculicerusella	658		Sami Haapala	02-Jun-2009	Finland	Savonia australis, Imatra	61,14	28,76		
MM19518	Elachista maculicerusella	307		H. Hendriksen	09-Aug-2004	Denmark	Nez, Sjaelland, Kulhuse	55,92	11,91		
MM21343	Elachista maculicerusella	658	M	J. Junnilainen	02-Jun-2008	Croatia	Jartsebarsko	45,67	15,65		
MM21425	Elachista maculosella	658	F	J. Nel	26-Apr-2003	France	Brusquires de Cassis	43,20	41399,00		
MM15561	Elachista nielswolffi	571	M	Marko Mutanen	11.-12.7.2000	Finland	Ostrobottnia borealis pars australis, Pudasjärvi	65,70	27,65		
MM16224	Elachista nielswolffi	658	M	J. Tabell	01-Jul-2009	Finland	Kainuu, Ostrobottnia kajanensis, Puolanka	64,65	28,02		
MM16225	Elachista nielswolffi	658	F	J. Tabell	ex larva em. 3-Jul-2008	Finland	Kainuu, Ostrobottnia kajanensis, Puolanka	64,65	28,05		Deschampsia flexuosa 24.VI.2008, GP 4429 JT
MM16226	Elachista nielswolffi	658	M	J. Tabell	01-Jul-2009	Finland	Kainuu, Ostrobottnia kajanensis, Puolanka	64,65	28,02		
MM17553	Elachista nielswolffi	658		Marko Mutanen	11.-12.7.2000	Finland	Ostrobottnia borealis pars australis, Pudasjärvi	65,70	27,65		
MM18151	Elachista nielswolffi	658		Marko Mutanen	11.-12.7.2000	Finland	Ostrobottnia borealis pars australis, Pudasjärvi	65,70	27,65		
MM20883	Elachista nielswolffi	407	M	T. Nupponen, K. Nupponen	24-Jun-2000	Russia	Altai Krai, Altai Mts., Sarlyk region	51,00	85,58	1450	
MM20885	Elachista nielswolffi	658	M	T. Nupponen, K. Nupponen	24-Jun-2000	Russia	Altai Krai, Altai Mts., Sarlyk region	51,00	85,58	1450	
MM20888	Elachista nielswolffi	658	M	T. Nupponen, K. Nupponen	24-Jun-2000	Russia	Altai Krai, Altai Mts., Sarlyk region	51,00	85,58	1450	

MM06159	Elachista nobilella	658		Marko Mutanen, Tomi Mutanen	18.-17.6.2007	Finland	Karelia ladogensis, Parikkala	61,64	29,64		
MM06160	Elachista nobilella	626[1n]		Marko Mutanen, Tomi Mutanen	18.-17.6.2007	Finland	Karelia ladogensis, Parikkala	61,64	29,64		
MM06267	Elachista nobilella	658	M	Marko Mutanen, Nestori Mutanen, Anttoni Mutanen	22.-23.6.2007	Finland	Ostrobottnia borealis pars australis, Kiiminki	65,07	25,73		
MM08381	Elachista nobilella	658		Marko Mutanen, Panu Vaelimaeki	17.-18.6.2005	Finland	Tavastia australis, Loppi	60,65	24,08		
MM17962	Elachista nobilella	658		Marko Mutanen, Nestori Mutanen, Anttoni Mutanen	19-Jun-2010	Finland	Karelia borealis, Kitee	62,08	29,95		
MM20033	Elachista nobilella	658	M	J. Tabell	01-Jul-2009	Finland	Kainuu, Ostrobottnia kajanensis, Puolanka	64,65	28,04		
MM20034	Elachista nobilella	658	M	J. Tabell	13-May-2007	Finland	Tavastia australis, Hartola	61,44	25,98		
MM16133	Elachista orstadii	658	M	J. Jalava, J. Kullberg	17-Jun-1995	Russia	Tyva, Tannu-Ola Mts	50,83	94,32		L. Kaila prep. 1836
MM16136	Elachista orstadii	658	M	O. Bidzilya	15-Jul-1995	Russia	Altai Krai, Ukok Plateau	49,30	87,50		
MM16137	Elachista orstadii	656	M	O. Bidzilya	18-Jun-1995	Russia	Altai Krai, Ukok Plateau	49,30	87,50		
MM18552	Elachista orstadii	658		Ingvar Svensson	15-May-1996	Sweden	Oeg, Heda	58,28	14,69		
MM18553	Elachista orstadii	658		Ingvar Svensson	02-Jun-1994	Sweden	Oel, Gardby	56,59	16,64		
MM18586	Elachista orstadii	658		Bengt A. Bengtsson	20-May-2010	Sweden	Sm, Hoegsby, Valakra	57,20	15,98		
MM18593	Elachista orstadii	658		Bengt A. Bengtsson	05-Jun-2004	Sweden	Oel, Gardby	56,59	16,64		
MM20879	Elachista orstadii	658	M	T. Nupponen, K. Nupponen	27-Jun-2000	Russia	Altai Krai, Altai Mts., Kuraisky hrebet	50,30	87,88	2750	
MM20882	Elachista orstadii	658	M	T. Nupponen, K. Nupponen	27-Jun-2000	Russia	Altai Krai, Altai Mts., Kuraisky hrebet	50,30	87,88	2750	
MM20884	Elachista orstadii	658	F	T. Nupponen, K. Nupponen	27-Jun-2000	Russia	Altai Krai, Altai Mts., Kuraisky hrebet	50,30	87,88	2750	
MM20900	Elachista orstadii	658	M	K. Nupponen	20-May-2004	Russia	Orenburg, South Ural, Guberlinsky Mts., Orsk 40 km W, near Guberlja village	51,25	58,08	340	
MM20901	Elachista orstadii	379	F	K. Nupponen	20-May-2004	Russia	Orenburg, South Ural, Guberlinsky Mts., Orsk 40 km W, near Guberlja village	51,25	58,08	340	
MM20906	Elachista orstadii	658	M	K. Nupponen	20-May-2004	Russia	Orenburg, South Ural, Guberlinsky Mts., Orsk 40 km W, near Guberlja village	51,25	58,08	340	
MM20970	Elachista orstadii	307	M	Timo Nupponen, Kari Nupponen	04-Jul-2000	Russia	Altai Krai, Altai mnts., Kuraisky hrebet	50,27	87,83	2500	
MM21452	Elachista orstadii	658	M	O. Karsholt	27-May-1994	Greece	Epirus, Ipiros, S. Metsova	39,75	21,19	1550	
MM21453	Elachista orstadii	658	M	O. Karsholt	27-May-1994	Greece	Epirus, Ipiros, S. Metsova	39,75	21,19	1550	
MM21454	Elachista orstadii	658	M	O. Karsholt	27-May-1994	Greece	Epirus, Ipiros, S. Metsova	39,75	21,19	1550	
MM15558	Elachista poae	658	M	Marko Mutanen	21-Jun-2004	Finland	Nylandia, Karjaa	60,19	23,75		

MM15559	Elachista poae	658	F	Marko Mutanen	21-Jun-2004	Finland	Nylandia, Karjaa	60,19	23,75		
MM16313	Elachista poae	658	M	J. Tabell	ex pupa 18.6.2008	Finland	Tavastia australis, Heinola	61,20	26,01		Glyceria maxima 12.VI.2008
MM02664	Elachista pomerana	658		Marko Mutanen, Panu Vaelimaeki		Finland	Karelia australis, Imatra	61,11	28,80		
MM02665	Elachista pomerana	651		Marko Mutanen, Panu Vaelimaeki		Finland	Karelia australis, Imatra	61,11	28,80		
MM02666	Elachista pomerana	658		Marko Mutanen, Panu Vaelimaeki	2006	Finland	Savonia australis, Imatra	61,11	28,80		
MM08066	Elachista pomerana	658		Marko Mutanen, Panu Vaelimaeki	3.-17.7.2006	Finland	Lapponia kemensis pars occidentalis, Kolari	67,28	23,75		
MM08634	Elachista pomerana	658	M	Marko Mutanen	18-Jul-2008	Finland	Aland Islands, Eckerö	60,28	19,58		
MM08635	Elachista pomerana	658	M	Marko Mutanen	18-Jul-2008	Finland	Aland Islands, Eckerö	60,28	19,58		
MM08636	Elachista pomerana	658	M	Marko Mutanen	18-Jul-2008	Finland	Aland Islands, Eckerö	60,28	19,58		
MM10299	Elachista pomerana	638		Marko Mutanen	ex larva June 2008	Finland	Ostrobottnia borealis pars australis, Kiiminki	65,07	25,73		larva on Calamagrostis purpurea
MM12474	Elachista pomerana	658		Marko Mutanen, Panu Vaelimaeki	2007	Finland	Savonia australis, Imatra	61,11	28,80		
MM12478	Elachista pomerana	658		Marko Mutanen, Panu Vaelimaeki	2007	Finland	Savonia australis, Imatra	61,11	28,80		
MM12479	Elachista pomerana	658		Marko Mutanen, Panu Vaelimaeki	2007	Finland	Savonia australis, Imatra	61,11	28,80		
MM12480	Elachista pomerana	658		Marko Mutanen, Panu Vaelimaeki	2007	Finland	Savonia australis, Imatra	61,11	28,80		
MM12481	Elachista pomerana	658		Marko Mutanen, Panu Vaelimaeki	2007	Finland	Savonia australis, Imatra	61,11	28,80		
MM13866	Elachista pomerana	427		M. Mutanen		Finland	Lapland, Tornio	65,90	24,45		
MM13867	Elachista pomerana	658		M. Mutanen		Finland	Lapland, Tornio	65,90	24,45		
MM14239	Elachista pomerana	658		Marko Mutanen, Nestori Mutanen, Anttoni Mutanen	29-Jun-2009	Finland	Aland Islands, Eckerö	60,24	19,59		
MM14642	Elachista pomerana	658		Marko Mutanen, Nestori Mutanen, Anttoni Mutanen	27-Jul-2009	Finland	Ostrobottnia borealis pars australis, Kiiminki	65,07	25,73		
MM16007	Elachista pomerana	658		Vesa Lepistoe, Kari Vaalamo	02-Jun-2006	Finland	Nylandia, Sipoo	60,22	25,38		
MM20014	Elachista pomerana	657[1n]	F	J. Tabell	02-Jul-2008	Finland	Tavastia australis, Hartola	61,57	26,05		
MM20909	Elachista pomerana	658	M	Jalava, Kullberg	13-Jul-1996	Russia	Buryatiya, Buryatia, Svyatov Nos pns., Monahovo	53,67	109,00	460	
MM18885	Elachista rufocinerea	658		P. Faiallo	04-May-1995	Italy	Liguria, Passo Turchino	44,48	8,73		
MM19517	Elachista rufocinerea	223		H. Hendriksen	03-May-1995	Denmark	Jylland, Hald ege	56,44	9,40		
MM21348	Elachista rufocinerea	658	M	L. Srnka	22-Mar-2001	Croatia	Zaostrog	43,14	17,27		
MM15299	Elachista subnigrella	658	M	A. Albrecht	02-Jun-1990	Estonia	Saaremaa, Mustjala	58,46	22,23		
MM16131	Elachista subnigrella	554	M	J. Bjorklund	14-Jun-1994	Sweden	Upland, Sollentuna	59,43	17,93		
MM16132	Elachista subnigrella	658	M	J. Bjorklund	14-Jun-1994	Sweden	Upland, Sollentuna	59,43	17,93		

MM16245	Elachista subnigrella	658	M	J. Tabell	ex larva em. 26-Jun-2005	Austria	Villach	46,61	13,85		GP 4515 L. Kaila
MM18550	Elachista subnigrella	658		Ingvar Svensson	03-Jun-2004	Sweden	Oel, Sandvik	57,07	16,86		
MM18551	Elachista subnigrella	658		Ingvar Svensson	17-Jun-1997	Sweden	Oeg, Styra	58,40	15,00		
MM19866	Elachista subnigrella	658		Ingvar Svensson	01-Jun-2004	Sweden	Oland, Lilla Horn	57,08	16,90		
MM21429	Elachista talgarella	658	M	L. Kaila	07-Aug-1990	Kazakhstan	Almaty, Transili Alatau, , Almaty Nat. Pk.	43,50	77,15	3000	paratype
MM21430	Elachista talgarella	540	M	L. Kaila	07-Aug-1990	Kazakhstan	Almaty, Transili Alatau, , Almaty Nat. Pk.	43,50	77,15	3000	paratype
MM16154	Elachista tanaella	658	M	L. Aarvik	15-Jul-2002	Norway	Fn, Tana Faccabaeljakka	70,30	28,20		paratype
TLMF Lep 04548	Elachista wieseriella	658		Huemer P.& Erlebach S.& Wieser	12-Jun-1999	Austria	Karnten, Auf der Mussen SE/ St.Jakob im Lesachtal N	46,71	12,94	1700	
TLMF Lep 06858	Elachista wieseriella	658		Deutsch H.	27-Jul-2005	Austria	Tirol, Osttirol, Koednitztal, Greiwiesen E/ Kals am Grossglockner	47,02	12,68	2100	
MM06515	Elachista vonschantzi	643	F	Marko Mutanen, Nestori Mutanen	03-Jul-2007	Finland	Ostrobottnia borealis pars australis, Hailuoto	65,05	24,88		
MM06516	Elachista vonschantzi	658	F	Marko Mutanen, Nestori Mutanen	03-Jul-2007	Finland	Ostrobottnia borealis pars australis, Hailuoto	65,05	24,88		
MM08614	Elachista vonschantzi	658	M	Marko Mutanen	30-Jun-2008	Finland	Ostrobottnia borealis pars australis, Hailuoto	65,05	24,88		
MM08615	Elachista vonschantzi	637	M	Marko Mutanen	30-Jun-2008	Finland	Ostrobottnia borealis pars australis, Hailuoto	65,05	24,88		
MM14167	Elachista vonschantzi	658		Marko Mutanen	24-Jun-2009	Finland	Ostrobottnia borealis pars australis, Hailuoto	65,03	24,88		
MM14168	Elachista vonschantzi	658		Marko Mutanen	24-Jun-2009	Finland	Ostrobottnia borealis pars australis, Hailuoto	65,03	24,88		
MM16121	Elachista zernyi	658	M	J. Jalava, J. Kullberg	20.-22.6.1995	Russia	W-Sajan Mts., Krasnojarsk Reg.	52,78	93,30		L. Kaila prep. 1837
MM16122	Elachista zernyi	623	M	J. Jalava, J. Kullberg	20.-22.6.1995	Russia	W-Sajan Mts., Krasnojarsk Reg.	52,78	93,30		
MM16123	Elachista zernyi	658	M	J. Jalava, J. Kullberg	20.-22.6.1995	Russia	W-Sajan Mts., Krasnojarsk Reg.	52,78	93,30		L. Kaila prep. 2843
MM16124	Elachista zernyi	658	F	J. Jalava, J. Kullberg	03-Jul-1996	Russia	Buryatiya, Barguzin Valley	54,83	111,3 0		
MM16125	Elachista zernyi	658	M	J. Jalava, J. Kullberg	4.-6.7.1996	Russia	Buryatiya, Barguzin Range	54,87	110,9 2		
MM17654	Elachista zernyi	658		Jari Kaitila	22-Jul-2000	Finland	Lapponia enontekiensis, Enontekiö	68,99	20,50		
MM18554	Elachista zernyi	658		Ingvar Svensson	02-Jul-1995	Sweden	Hr, Hamrafjaellet	62,56	12,29		
MM18555	Elachista zernyi	658		Ingvar Svensson	01-Jul-1995	Sweden	Hr, Hamrafjaellet	62,56	12,29		
MM20931	Elachista zernyi	658	M	K. Nupponen	17-Jun-2002	Russia	Irkutsk, Irkutsk prov., Sludjanka village 2 km SE, Lake Baikal shope slope	51,67	103,7 2	500	

MM20932	Elachista zernyi	407	M	K. Nupponen	16-Jun-2002	Russia	Buryatiya, SW-Buryatia, East Sayan Mts., Halagun river, Turan village 8 km W	51,64	101,58	900	
MM20933	Elachista zernyi	658	M	K. Nupponen	15-Jun-2002	Russia	Buryatiya, SW-Buryatia, East Sayan Mts., Mondy village 2 km E	51,68	101,02	1450	
MM22606	Elachista zernyi	658	M	J.-P. Kaitila	12-Jul-2008	Finland	Lapponia enontekiensis, Enontekiö, Toshkaljoki	69,16	21,58		
MM16120	Elachista sp. 01MM	658	M	K. Mikkola	14-Jul-1991	Russia	Chukot Autonomous Okrug, Chuckhi Pns, Provideniya	64,92	172,50		BAB prep. 396X
MM16145	Elachista sp. 02MM	658	M	J. Jalava, J. Kullberg	04-Jul-1994	Russia	Polar Ural, Europe Asia Pass	67,03	65,08		L. Kaila prep. 1208
MM16146	Elachista sp. 02MM	639	M	J. Jalava, J. Kullberg	04-Jul-1994	Russia	Polar Ural, Europe Asia Pass	67,03	65,08		L. Kaila prep. 4855
MM16181	Elachista sp. 03MM	551[1n]	M	Jalava, Kullberg, Koponen	02-Jul-1994	Russia	Polar Ural, Krasnyi Kamen	66,08	65,17		L.Kaila prep. 1209
MM21420	Elachista sp. 03MM	658	M	Jalava, Kullberg, Koponen	02-Jul-1994	Russia	Polar Ural, Krasnyi Kamen	66,55	65,10	200	
MM21421	Elachista sp. 04MM	627	M	L. Kaila	31-Jul-1990	Kyrgyzstan	Naryn, 45 km NE Naryn	41,40	76,31	2650	
MM21377	Elachista sp. 05MM	658	M	Z. Tokar	07-Jul-2000	Slovenia	Juliske Alpe, Mangart	46,43	13,65	2000	
MM21382	Elachista sp. 05MM	658	M	Z. Tokar	07-Jul-2000	Slovenia	Juliske Alpe, Mangart	46,43	13,65	2000	
MM21476	Elachista sp. 06MM	658	M	K. Nupponen, R. Haverinen	23-Jul-2010	Kyrgyzstan	Alai mts, Tengiz-Bai pass	39,38	72,14	3650	
MM21477	Elachista sp. 06MM	650	M	K. Nupponen, R. Haverinen	23-Jul-2010	Kyrgyzstan	Alai mts, Tengiz-Bai pass	39,38	72,14	3650	
MM16170	Elachista sp. 07MM	636	M	Yu. Budashkin	19-May-1996	Ukraine	Krym, Karadagh	44,90	35,20		
MM21388	Elachista sp. 07MM	658	F	Yu. Budashkin	20-May-1996	Ukraine	Crimea, Karadagh	44,90	35,20		
MM21389	Elachista sp. 07MM	658	F	Yu. Budashkin	28-May-1996	Ukraine	Crimea, Karadagh	44,90	35,20		
MM21391	Elachista sp. 07MM	253	F	Yu. Budashkin	12-Jun-1994	Ukraine	Crimea, Karadagh	44,90	35,20		
MM20908	Elachista sp. 08MM	658	M	Jalava, Tammaru	08-Jul-1997	Russia	Magadan, Magadanskaya Oblast, Magadan 15 km E	59,57	151,20	50	
MM20914	Elachista sp. 08MM	658	M	Jalava, Tammaru	08-Jul-1997	Russia	Magadan, Magadanskaya Oblast, Magadan 15 km E	59,57	151,20	50	
MM20915	Elachista sp. 08MM	658	F	Jalava, Tammaru	07-Jul-1997	Russia	Magadan, Magadanskaya Oblast, Magadan 15 km E	59,57	151,20	50	
MM20916	Elachista sp. 08MM	658	M	Jalava, Tammaru	07-Jul-1997	Russia	Magadan, Magadanskaya Oblast, Magadan 15 km E	59,57	151,20	50	
MM20917	Elachista sp. 08MM	658	M	Jalava, Tammaru	07-Jul-1997	Russia	Magadan, Magadanskaya Oblast, Magadan 15 km E	59,57	151,20	50	
MM20857	Elachista sp. 09MM	658	F	O. Karsholt	27-May-1994	Greece	Epirus, Ipiros, S. Metsova	39,75	21,19	1550	
MM21455	Elachista sp. 09MM	626	M	O. Karsholt	27-May-1994	Greece	Epirus, Ipiros, S. Metsova	39,75	21,19	1550	

MM20892	Elachista sp. 10MM	658	M	T. Nupponen, K. Nupponen	25-Jun-2000	Russia	Altai Krai, Altai Mts., Kuraisky steppe	50,25	87,88	1600	
MM20880	Elachista sp. 11MM	658	M	T. Nupponen, K. Nupponen	27-Jun-2000	Russia	Altai Krai, Altai Mts., Kuraisky hrebet	50,30	87,88	2750	
MM20893	Elachista sp. 11MM	658	M	K. Nupponen	08-Jul-2001	Russia	Altai Krai, Altai Mts., Kuraisky hrebet	50,30	87,88	2300	
MM20894	Elachista sp. 11MM	658	F	K. Nupponen	08-Jul-2001	Russia	Altai Krai, Altai Mts., Kuraisky hrebet	50,30	87,88	2300	
MM20967	Elachista sp. 11MM	658	M	K. Nupponen	07-Jul-2001	Russia	Altai Krai, Altai mnts., Kuraisky hrebet	50,27	87,83	2300	
MM20972	Elachista sp. 11MM	658	M	K. Nupponen	11-Jul-2001	Russia	Altai Krai, Altai mnts., Kuraisky hrebet	50,27	87,83	2300	
MM20878	Elachista sp. 12MM	658	M	T. Nupponen, K. Nupponen	24-Jun-2000	Russia	Altai Krai, Altai Mts., Sarlyk region	51,00	85,58	1450	
MM20886	Elachista sp. 12MM	658	F	T. Nupponen, K. Nupponen	24-Jun-2000	Russia	Altai Krai, Altai Mts., Sarlyk region	51,00	85,58	1450	
MM20968	Elachista sp. 12MM	658	M	K. Nupponen	10-Jul-2001	Russia	Altai Krai, Altai mnts., Kuraisky hrebet	50,27	87,83	2300	
MM20969	Elachista sp. 12MM	658	M	K. Nupponen	09-Jul-2001	Russia	Altai Krai, Altai mnts., Kuraisky hrebet	50,27	87,83	2300	
MM20971	Elachista sp. 12MM	658	M	K. Nupponen	07-Jul-2001	Russia	Altai Krai, Altai mnts., Kuraisky hrebet	50,27	87,83	2300	
MM20973	Elachista sp. 12MM	658	M	K. Nupponen	09-Jul-2001	Russia	Altai Krai, Altai mnts., Kuraisky hrebet	50,27	87,83	2300	
MM21468	Elachista sp. 12MM	658	M	K. Nupponen	11-Jul-2001	Russia	Altai Krai, Altai Mts, Kuraisky hrebet	50,16	87,50	2300	
MM21470	Elachista sp. 12MM	658	M	K. Nupponen	13-Jul-2001	Russia	Altai Krai, Altai Mts, Kuraisky hrebet	50,16	87,50	2300	
MM21472	Elachista sp. 12MM	658	F	K. Nupponen	06-Jul-2001	Russia	Altai Krai, Altai Mts, Kuraisky hrebet	50,16	87,50	2300	
MM21473	Elachista sp. 12MM	411	M	K. Nupponen	07-Jul-2001	Russia	Altai Krai, Altai Mts, Kuraisky hrebet	50,16	87,50	2300	
MM21474	Elachista sp. 12MM	658	M	K. Nupponen	10-Jul-2001	Russia	Altai Krai, Altai Mts, Kuraisky hrebet	50,16	87,50	2300	
MM21475	Elachista sp. 12MM	406[2n]	M	K. Nupponen	07-Jul-2001	Russia	Altai Krai, Altai Mts, Kuraisky hrebet	50,16	87,50	2300	
MM22535	Elachista sp. 12MM	658	M	J. Jalava, J. Kullberg	17-Jun-1995	Russia	Tyva, Tannu-Ola Mts	50,50	94,19	2175	
MM22536	Elachista sp. 12MM	658	M	J. Jalava, J. Kullberg	17-Jun-1995	Russia	Tyva, Tannu-Ola Mts	50,50	94,19	2175	
MM22541	Elachista sp. 13MM	658	M	K. Nupponen	05-Aug-2010	Kyrgyzstan	Tien-Shan Mts., Eki-Naryn	41,31	76,30	2600	
MM15492	Elachista sp. 14MM	658	M	K, Nupponen, J. Junnilainen	06-May-1996	Turkey	Aksehir, Sultan Daglari	38,33	31,33		L. Kaila prep. 3017
MM15493	Elachista sp. 14MM	658	M	T. Nupponen	09-Jun-2002	Turkey	Aksehir, Sultan Daglari	38,33	31,33		
MM15494	Elachista sp. 14MM	658	M	J. Junnilainen	9.-13.5.2000	Turkey	Aksehir, Cetince	38,18	31,21		
MM21341	Elachista sp. 14MM	259	M	J. Junnilainen	20-May-2005	Turkey	Aksehir, Sultan Daglari	38,33	31,33		
MM21649	Elachista sp. 15MM	658	M	J. Tabell	02-May-2012	Greece	Crete, Omalos 2 km NNE	35,34	23,90	1030	
MM22599	Elachista sp. 16MM	658	M	K. Nupponen, J. Junnilainen	26-Jun-1997	Russia	Chelyabinsk, South Ural, Miass town, Ilmen State Reserve	55,01	60,10	350	

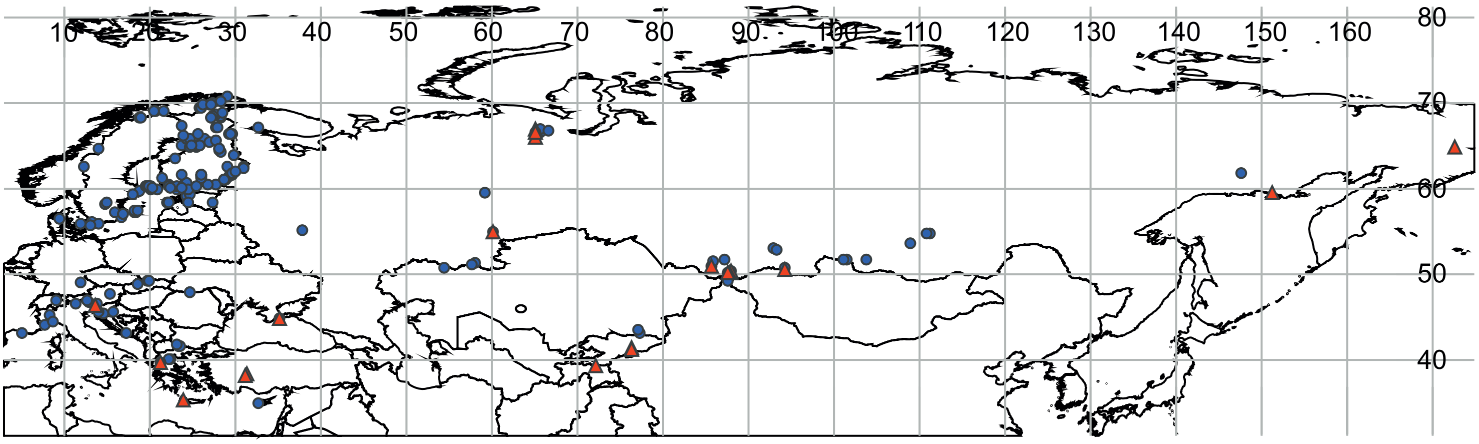

Figure S1. A map of collection localities of the samples included in this study. Records representing named species are depicted as blue dots, records representing putative new species as red triangles. Each of the 16 putative new species was found only in a single locality. The map was created using the SimpleMappr[64].

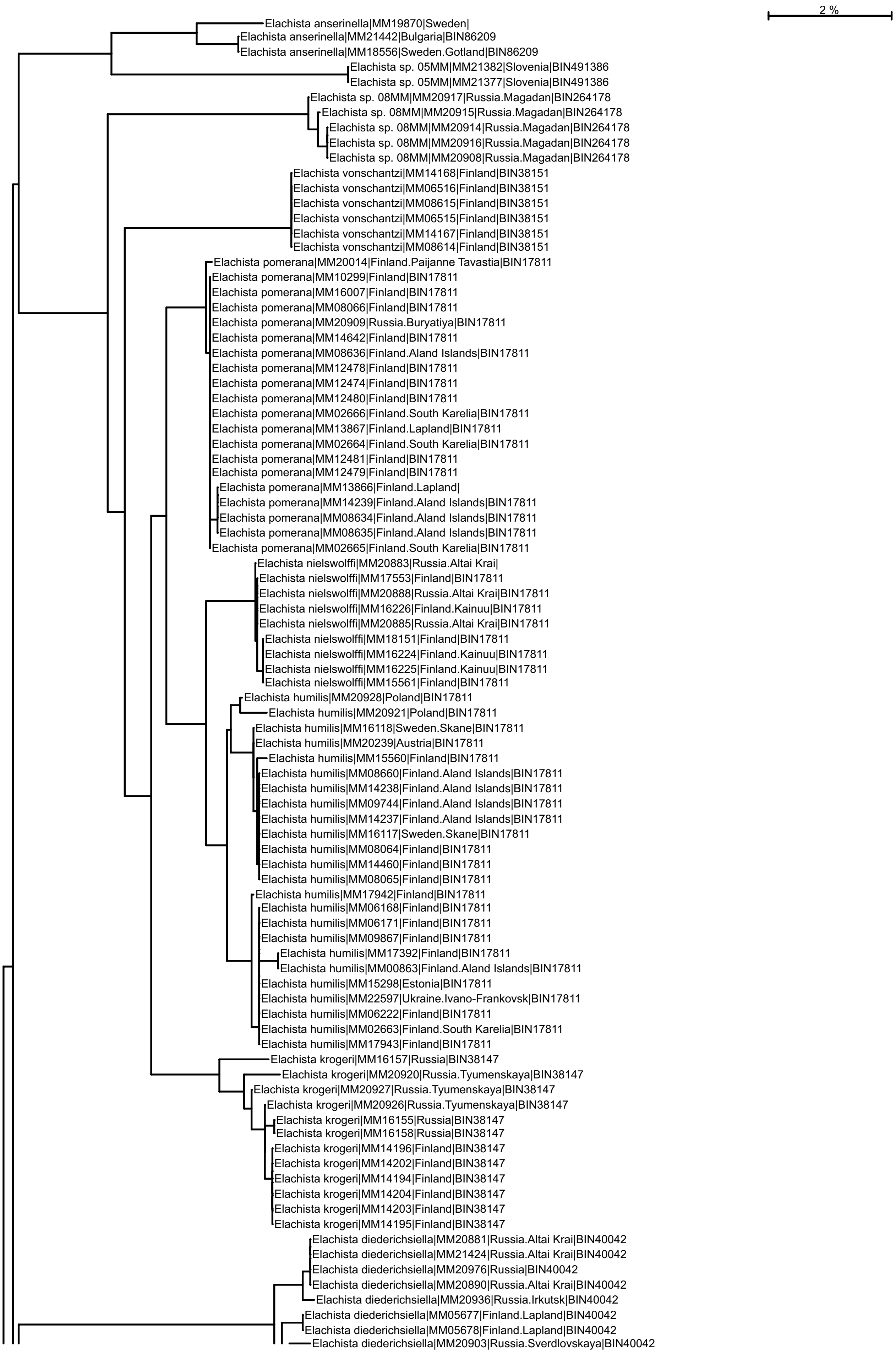
2 %
Elachista anserinella|MM19870|Sweden|
Elachista anserinella|MM21442|Bulgaria|BIN86209
Elachista anserinella|MM18556|Sweden.Gotland|BIN86209
Elachista sp. 05MM|MM21382|Slovenia|BIN491386
Elachista sp. 05MM|MM21377|Slovenia|BIN491386
Elachista sp. 08MM|MM20917|Russia.Magadan|BIN264178
Elachista sp. 08MM|MM20915|Russia.Magadan|BIN264178
Elachista sp. 08MM|MM20914|Russia.Magadan|BIN264178
Elachista sp. 08MM|MM20916|Russia.Magadan|BIN264178
Elachista sp. 08MM|MM20908|Russia.Magadan|BIN264178
Elachista vonschantzi|MM14168|Finland|BIN38151
Elachista vonschantzi|MM06516|Finland|BIN38151
Elachista vonschantzi|MM08615|Finland|BIN38151
Elachista vonschantzi|MM06515|Finland|BIN38151
Elachista vonschantzi|MM14167|Finland|BIN38151
Elachista vonschantzi|MM08614|Finland|BIN38151
Elachista pomerana|MM20014|Finland.Paijanne Tavastia|BIN17811
Elachista pomerana|MM10299|Finland|BIN17811
Elachista pomerana|MM16007|Finland|BIN17811
Elachista pomerana|MM08066|Finland|BIN17811
Elachista pomerana|MM20909|Russia.Buryatiya|BIN17811
Elachista pomerana|MM14642|Finland|BIN17811
Elachista pomerana|MM08636|Finland.Aland Islands|BIN17811
Elachista pomerana|MM12478|Finland|BIN17811
Elachista pomerana|MM12474|Finland|BIN17811
Elachista pomerana|MM12480|Finland|BIN17811
Elachista pomerana|MM02666|Finland.South Karelia|BIN17811
Elachista pomerana|MM13867|Finland.Lapland|BIN17811
Elachista pomerana|MM02664|Finland.South Karelia|BIN17811
Elachista pomerana|MM12481|Finland|BIN17811
Elachista pomerana|MM12479|Finland|BIN17811
Elachista pomerana|MM13866|Finland.Lapland|
Elachista pomerana|MM14239|Finland.Aland Islands|BIN17811
Elachista pomerana|MM08634|Finland.Aland Islands|BIN17811
Elachista pomerana|MM08635|Finland.Aland Islands|BIN17811
Elachista pomerana|MM02665|Finland.South Karelia|BIN17811
Elachista nielswolffi|MM20883|Russia.Altai Krai|
Elachista nielswolffi|MM17553|Finland|BIN17811
Elachista nielswolffi|MM20888|Russia.Altai Krai|BIN17811
Elachista nielswolffi|MM16226|Finland.Kainuu|BIN17811
Elachista nielswolffi|MM20885|Russia.Altai Krai|BIN17811
Elachista nielswolffi|MM18151|Finland|BIN17811
Elachista nielswolffi|MM16224|Finland.Kainuu|BIN17811
Elachista nielswolffi|MM16225|Finland.Kainuu|BIN17811
Elachista nielswolffi|MM15561|Finland|BIN17811
Elachista humilis|MM20928|Poland|BIN17811
Elachista humilis|MM20921|Poland|BIN17811
Elachista humilis|MM16118|Sweden.Skane|BIN17811
Elachista humilis|MM20239|Austria|BIN17811
Elachista humilis|MM15560|Finland|BIN17811
Elachista humilis|MM08660|Finland.Aland Islands|BIN17811
Elachista humilis|MM14238|Finland.Aland Islands|BIN17811
Elachista humilis|MM09744|Finland.Aland Islands|BIN17811
Elachista humilis|MM14237|Finland.Aland Islands|BIN17811
Elachista humilis|MM16117|Sweden.Skane|BIN17811
Elachista humilis|MM08064|Finland|BIN17811
Elachista humilis|MM14460|Finland|BIN17811
Elachista humilis|MM08065|Finland|BIN17811
Elachista humilis|MM17942|Finland|BIN17811
Elachista humilis|MM06168|Finland|BIN17811
Elachista humilis|MM06171|Finland|BIN17811
Elachista humilis|MM09867|Finland|BIN17811
Elachista humilis|MM17392|Finland|BIN17811
Elachista humilis|MM00863|Finland.Aland Islands|BIN17811
Elachista humilis|MM15298|Estonia|BIN17811
Elachista humilis|MM22597|Ukraine.Ivano-Frankovsk|BIN17811
Elachista humilis|MM06222|Finland|BIN17811
Elachista humilis|MM02663|Finland.South Karelia|BIN17811
Elachista humilis|MM17943|Finland|BIN17811
Elachista krogeri|MM16157|Russia|BIN38147
Elachista krogeri|MM20920|Russia.Tyumenskaya|BIN38147
Elachista krogeri|MM20927|Russia.Tyumenskaya|BIN38147
Elachista krogeri|MM20926|Russia.Tyumenskaya|BIN38147
Elachista krogeri|MM16155|Russia|BIN38147
Elachista krogeri|MM16158|Russia|BIN38147
Elachista krogeri|MM14196|Finland|BIN38147
Elachista krogeri|MM14202|Finland|BIN38147
Elachista krogeri|MM14194|Finland|BIN38147
Elachista krogeri|MM14204|Finland|BIN38147
Elachista krogeri|MM14203|Finland|BIN38147
Elachista krogeri|MM14195|Finland|BIN38147
Elachista diederichsiella|MM20881|Russia.Altai Krai|BIN40042
Elachista diederichsiella|MM21424|Russia.Altai Krai|BIN40042
Elachista diederichsiella|MM20976|Russia|BIN40042
Elachista diederichsiella|MM20890|Russia.Altai Krai|BIN40042
Elachista diederichsiella|MM20936|Russia.Irkutsk|BIN40042
Elachista diederichsiella|MM05677|Finland.Lapland|BIN40042
Elachista diederichsiella|MM05678|Finland.Lapland|BIN40042
Elachista diederichsiella|MM20903|Russia.Sverdlovskaya|BIN40042

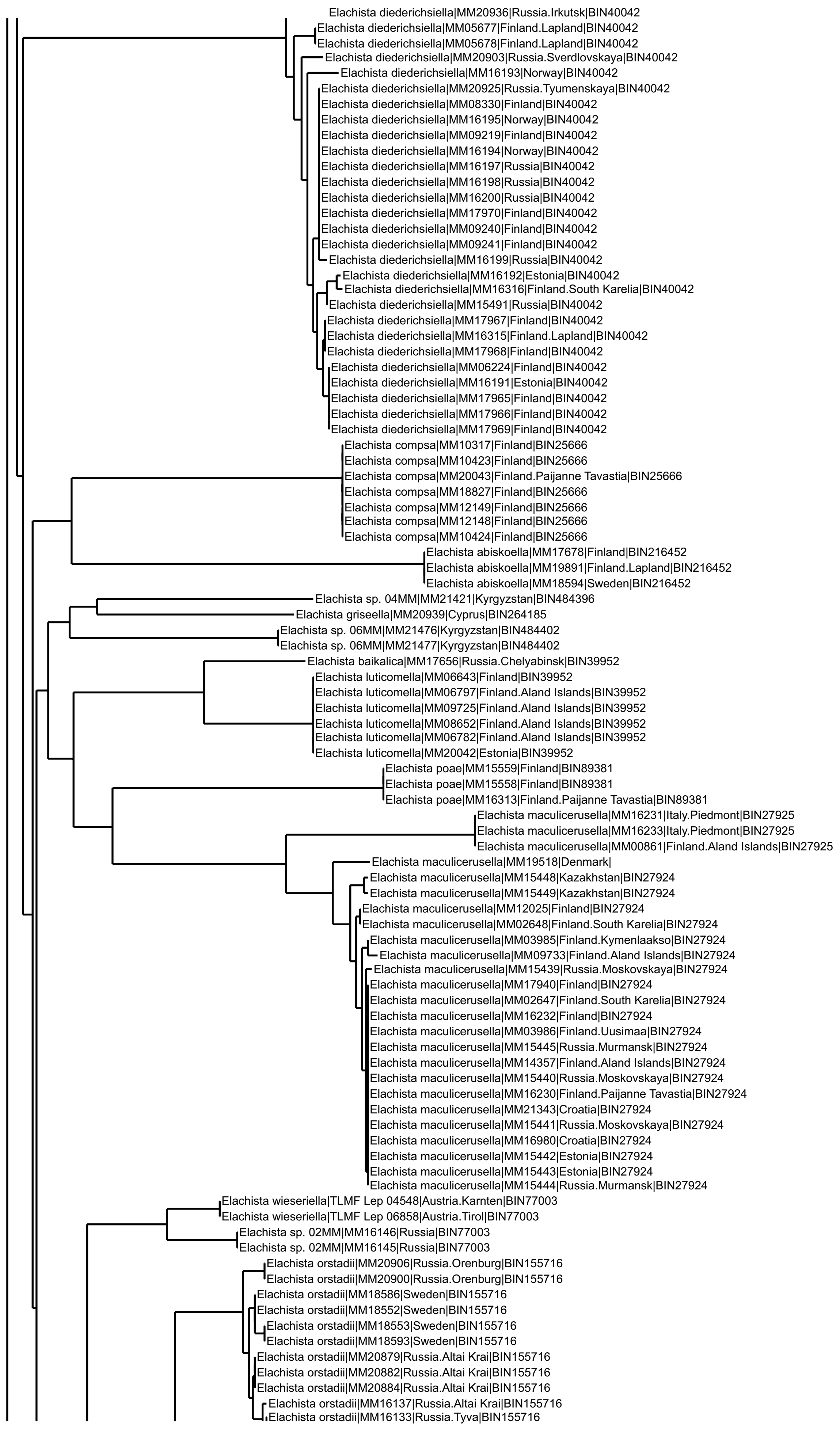

Elachista diederichsiella|MM20936|Russia.Irkutsk|BIN40042
Elachista diederichsiella|MM05677|Finland.Lapland|BIN40042
Elachista diederichsiella|MM05678|Finland.Lapland|BIN40042
Elachista diederichsiella|MM20903|Russia.Sverdlovskaya|BIN40042
Elachista diederichsiella|MM16193|Norway|BIN40042
Elachista diederichsiella|MM20925|Russia.Tyumenskaya|BIN40042
Elachista diederichsiella|MM08330|Finland|BIN40042
Elachista diederichsiella|MM16195|Norway|BIN40042
Elachista diederichsiella|MM09219|Finland|BIN40042
Elachista diederichsiella|MM16194|Norway|BIN40042
Elachista diederichsiella|MM16197|Russia|BIN40042
Elachista diederichsiella|MM16198|Russia|BIN40042
Elachista diederichsiella|MM16200|Russia|BIN40042
Elachista diederichsiella|MM17970|Finland|BIN40042
Elachista diederichsiella|MM09240|Finland|BIN40042
Elachista diederichsiella|MM09241|Finland|BIN40042
Elachista diederichsiella|MM16199|Russia|BIN40042
Elachista diederichsiella|MM16192|Estonia|BIN40042
Elachista diederichsiella|MM16316|Finland.South Karelia|BIN40042
Elachista diederichsiella|MM15491|Russia|BIN40042
Elachista diederichsiella|MM17967|Finland|BIN40042
Elachista diederichsiella|MM16315|Finland.Lapland|BIN40042
Elachista diederichsiella|MM17968|Finland|BIN40042
Elachista diederichsiella|MM06224|Finland|BIN40042
Elachista diederichsiella|MM16191|Estonia|BIN40042
Elachista diederichsiella|MM17965|Finland|BIN40042
Elachista diederichsiella|MM17966|Finland|BIN40042
Elachista diederichsiella|MM17969|Finland|BIN40042
Elachista compsa|MM10317|Finland|BIN25666
Elachista compsa|MM10423|Finland|BIN25666
Elachista compsa|MM20043|Finland.Paijanne Tavastia|BIN25666
Elachista compsa|MM18827|Finland|BIN25666
Elachista compsa|MM12149|Finland|BIN25666
Elachista compsa|MM12148|Finland|BIN25666
Elachista compsa|MM10424|Finland|BIN25666
Elachista abiskoella|MM17678|Finland|BIN216452
Elachista abiskoella|MM19891|Finland.Lapland|BIN216452
Elachista abiskoella|MM18594|Sweden|BIN216452
Elachista sp. 04MM|MM21421|Kyrgyzstan|BIN484396
Elachista griseella|MM20939|Cyprus|BIN264185
Elachista sp. 06MM|MM21476|Kyrgyzstan|BIN484402
Elachista sp. 06MM|MM21477|Kyrgyzstan|BIN484402
Elachista baikalica|MM17656|Russia.Chelyabinsk|BIN39952
Elachista luticomella|MM06643|Finland|BIN39952
Elachista luticomella|MM06797|Finland.Aland Islands|BIN39952
Elachista luticomella|MM09725|Finland.Aland Islands|BIN39952
Elachista luticomella|MM08652|Finland.Aland Islands|BIN39952
Elachista luticomella|MM06782|Finland.Aland Islands|BIN39952
Elachista luticomella|MM20042|Estonia|BIN39952
Elachista poae|MM15559|Finland|BIN89381
Elachista poae|MM15558|Finland|BIN89381
Elachista poae|MM16313|Finland.Paijanne Tavastia|BIN89381
Elachista maculicerusella|MM16231|Italy.Piedmont|BIN27925
Elachista maculicerusella|MM16233|Italy.Piedmont|BIN27925
Elachista maculicerusella|MM00861|Finland.Aland Islands|BIN27925
Elachista maculicerusella|MM19518|Denmark|
Elachista maculicerusella|MM15448|Kazakhstan|BIN27924
Elachista maculicerusella|MM15449|Kazakhstan|BIN27924
Elachista maculicerusella|MM12025|Finland|BIN27924
Elachista maculicerusella|MM02648|Finland.South Karelia|BIN27924
Elachista maculicerusella|MM03985|Finland.Kymenlaakso|BIN27924
Elachista maculicerusella|MM09733|Finland.Aland Islands|BIN27924
Elachista maculicerusella|MM15439|Russia.Moskovskaya|BIN27924
Elachista maculicerusella|MM17940|Finland|BIN27924
Elachista maculicerusella|MM02647|Finland.South Karelia|BIN27924
Elachista maculicerusella|MM16232|Finland|BIN27924
Elachista maculicerusella|MM03986|Finland.Uusimaa|BIN27924
Elachista maculicerusella|MM15445|Russia.Murmansk|BIN27924
Elachista maculicerusella|MM14357|Finland.Aland Islands|BIN27924
Elachista maculicerusella|MM15440|Russia.Moskovskaya|BIN27924
Elachista maculicerusella|MM16230|Finland.Paijanne Tavastia|BIN27924
Elachista maculicerusella|MM21343|Croatia|BIN27924
Elachista maculicerusella|MM15441|Russia.Moskovskaya|BIN27924
Elachista maculicerusella|MM16980|Croatia|BIN27924
Elachista maculicerusella|MM15442|Estonia|BIN27924
Elachista maculicerusella|MM15443|Estonia|BIN27924
Elachista maculicerusella|MM15444|Russia.Murmansk|BIN27924
Elachista wieseriella|TLMF Lep 04548|Austria.Karnten|BIN77003
Elachista wieseriella|TLMF Lep 06858|Austria.Tirol|BIN77003
Elachista sp. 02MM|MM16146|Russia|BIN77003
Elachista sp. 02MM|MM16145|Russia|BIN77003
Elachista orstadii|MM20906|Russia.Orenburg|BIN155716
Elachista orstadii|MM20900|Russia.Orenburg|BIN155716
Elachista orstadii|MM18586|Sweden|BIN155716
Elachista orstadii|MM18552|Sweden|BIN155716
Elachista orstadii|MM18553|Sweden|BIN155716
Elachista orstadii|MM18593|Sweden|BIN155716
Elachista orstadii|MM20879|Russia.Altai Krai|BIN155716
Elachista orstadii|MM20882|Russia.Altai Krai|BIN155716
Elachista orstadii|MM20884|Russia.Altai Krai|BIN155716
Elachista orstadii|MM16137|Russia.Altai Krai|BIN155716
Elachista orstadii|MM16133|Russia.Tyva|BIN155716

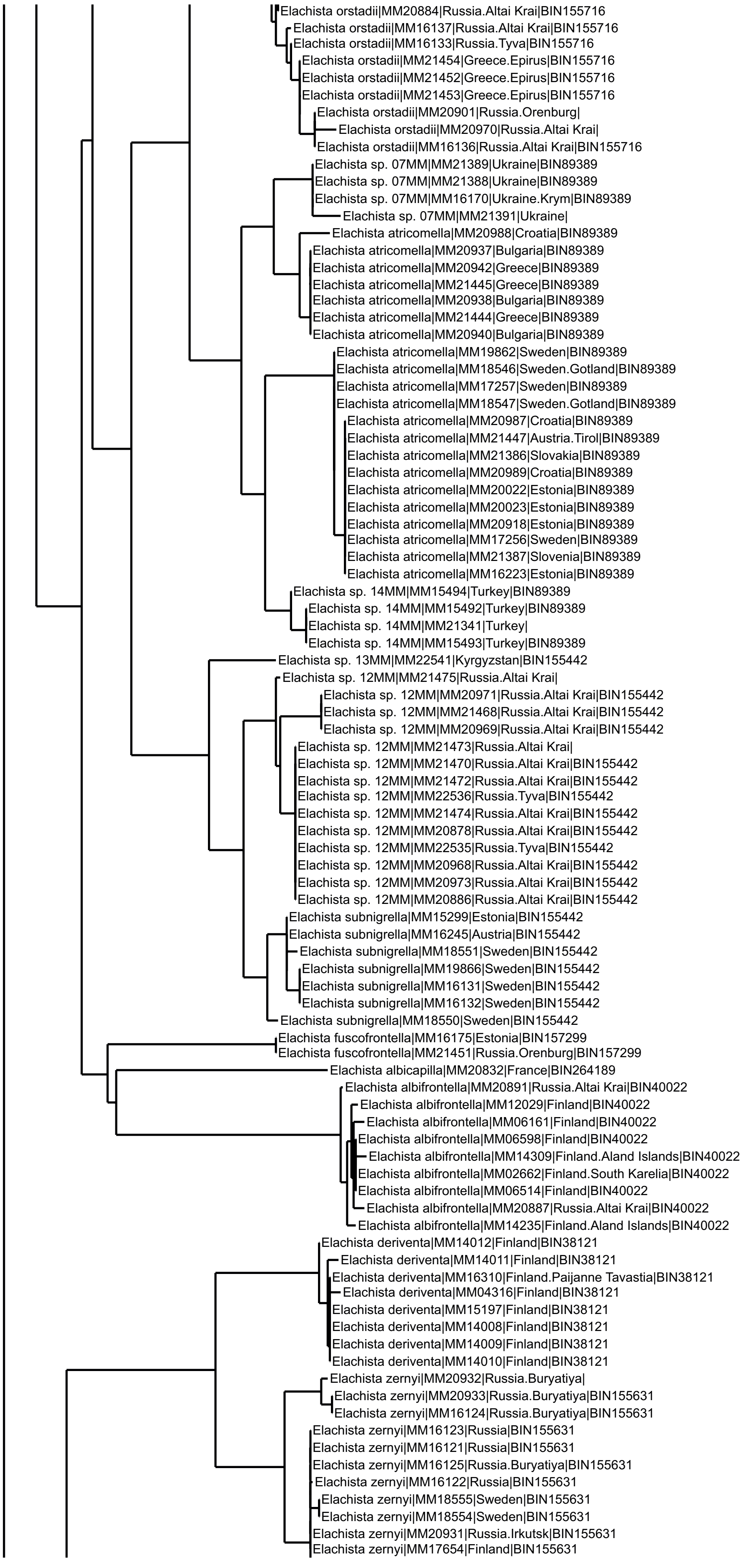

Elachista orstadii|MM20884|Russia.Altai Krai|BIN155716
Elachista orstadii|MM16137|Russia.Altai Krai|BIN155716
Elachista orstadii|MM16133|Russia.Tyva|BIN155716
Elachista orstadii|MM21454|Greece.Epirus|BIN155716
Elachista orstadii|MM21452|Greece.Epirus|BIN155716
Elachista orstadii|MM21453|Greece.Epirus|BIN155716
Elachista orstadii|MM20901|Russia.Orenburg|
Elachista orstadii|MM20970|Russia.Altai Krai|
Elachista orstadii|MM16136|Russia.Altai Krai|BIN155716
Elachista sp. 07MM|MM21389|Ukraine|BIN89389
Elachista sp. 07MM|MM21388|Ukraine|BIN89389
Elachista sp. 07MM|MM16170|Ukraine.Krym|BIN89389
Elachista sp. 07MM|MM21391|Ukraine|
Elachista atricomella|MM20988|Croatia|BIN89389
Elachista atricomella|MM20937|Bulgaria|BIN89389
Elachista atricomella|MM20942|Greece|BIN89389
Elachista atricomella|MM21445|Greece|BIN89389
Elachista atricomella|MM20938|Bulgaria|BIN89389
Elachista atricomella|MM21444|Greece|BIN89389
Elachista atricomella|MM20940|Bulgaria|BIN89389
Elachista atricomella|MM19862|Sweden|BIN89389
Elachista atricomella|MM18546|Sweden.Gotland|BIN89389
Elachista atricomella|MM17257|Sweden|BIN89389
Elachista atricomella|MM18547|Sweden.Gotland|BIN89389
Elachista atricomella|MM20987|Croatia|BIN89389
Elachista atricomella|MM21447|Austria.Tirol|BIN89389
Elachista atricomella|MM21386|Slovakia|BIN89389
Elachista atricomella|MM20989|Croatia|BIN89389
Elachista atricomella|MM20022|Estonia|BIN89389
Elachista atricomella|MM20023|Estonia|BIN89389
Elachista atricomella|MM20918|Estonia|BIN89389
Elachista atricomella|MM17256|Sweden|BIN89389
Elachista atricomella|MM21387|Slovenia|BIN89389
Elachista atricomella|MM16223|Estonia|BIN89389
Elachista sp. 14MM|MM15494|Turkey|BIN89389
Elachista sp. 14MM|MM15492|Turkey|BIN89389
Elachista sp. 14MM|MM21341|Turkey|
Elachista sp. 14MM|MM15493|Turkey|BIN89389
Elachista sp. 13MM|MM22541|Kyrgyzstan|BIN155442
Elachista sp. 12MM|MM21475|Russia.Altai Krai|
Elachista sp. 12MM|MM20971|Russia.Altai Krai|BIN155442
Elachista sp. 12MM|MM21468|Russia.Altai Krai|BIN155442
Elachista sp. 12MM|MM20969|Russia.Altai Krai|BIN155442
Elachista sp. 12MM|MM21473|Russia.Altai Krai|
Elachista sp. 12MM|MM21470|Russia.Altai Krai|BIN155442
Elachista sp. 12MM|MM21472|Russia.Altai Krai|BIN155442
Elachista sp. 12MM|MM22536|Russia.Tyva|BIN155442
Elachista sp. 12MM|MM21474|Russia.Altai Krai|BIN155442
Elachista sp. 12MM|MM20878|Russia.Altai Krai|BIN155442
Elachista sp. 12MM|MM22535|Russia.Tyva|BIN155442
Elachista sp. 12MM|MM20968|Russia.Altai Krai|BIN155442
Elachista sp. 12MM|MM20973|Russia.Altai Krai|BIN155442
Elachista sp. 12MM|MM20886|Russia.Altai Krai|BIN155442
Elachista subnigrella|MM15299|Estonia|BIN155442
Elachista subnigrella|MM16245|Austria|BIN155442
Elachista subnigrella|MM18551|Sweden|BIN155442
Elachista subnigrella|MM19866|Sweden|BIN155442
Elachista subnigrella|MM16131|Sweden|BIN155442
Elachista subnigrella|MM16132|Sweden|BIN155442
Elachista subnigrella|MM18550|Sweden|BIN155442
Elachista fuscofrontella|MM16175|Estonia|BIN157299
Elachista fuscofrontella|MM21451|Russia.Orenburg|BIN157299
Elachista albicapilla|MM20832|France|BIN264189
Elachista albifrontella|MM20891|Russia.Altai Krai|BIN40022
Elachista albifrontella|MM12029|Finland|BIN40022
Elachista albifrontella|MM06161|Finland|BIN40022
Elachista albifrontella|MM06598|Finland|BIN40022
Elachista albifrontella|MM14309|Finland.Aland Islands|BIN40022
Elachista albifrontella|MM02662|Finland.South Karelia|BIN40022
Elachista albifrontella|MM06514|Finland|BIN40022
Elachista albifrontella|MM20887|Russia.Altai Krai|BIN40022
Elachista albifrontella|MM14235|Finland.Aland Islands|BIN40022
Elachista deriventa|MM14012|Finland|BIN38121
Elachista deriventa|MM14011|Finland|BIN38121
Elachista deriventa|MM16310|Finland.Paijanne Tavastia|BIN38121
Elachista deriventa|MM04316|Finland|BIN38121
Elachista deriventa|MM15197|Finland|BIN38121
Elachista deriventa|MM14008|Finland|BIN38121
Elachista deriventa|MM14009|Finland|BIN38121
Elachista deriventa|MM14010|Finland|BIN38121
Elachista zernyi|MM20932|Russia.Buryatiya|
Elachista zernyi|MM20933|Russia.Buryatiya|BIN155631
Elachista zernyi|MM16124|Russia.Buryatiya|BIN155631
Elachista zernyi|MM16123|Russia|BIN155631
Elachista zernyi|MM16121|Russia|BIN155631
Elachista zernyi|MM16125|Russia.Buryatiya|BIN155631
Elachista zernyi|MM16122|Russia|BIN155631
Elachista zernyi|MM18555|Sweden|BIN155631
Elachista zernyi|MM18554|Sweden|BIN155631
Elachista zernyi|MM20931|Russia.Irkutsk|BIN155631
Elachista zernyi|MM17654|Finland|BIN155631

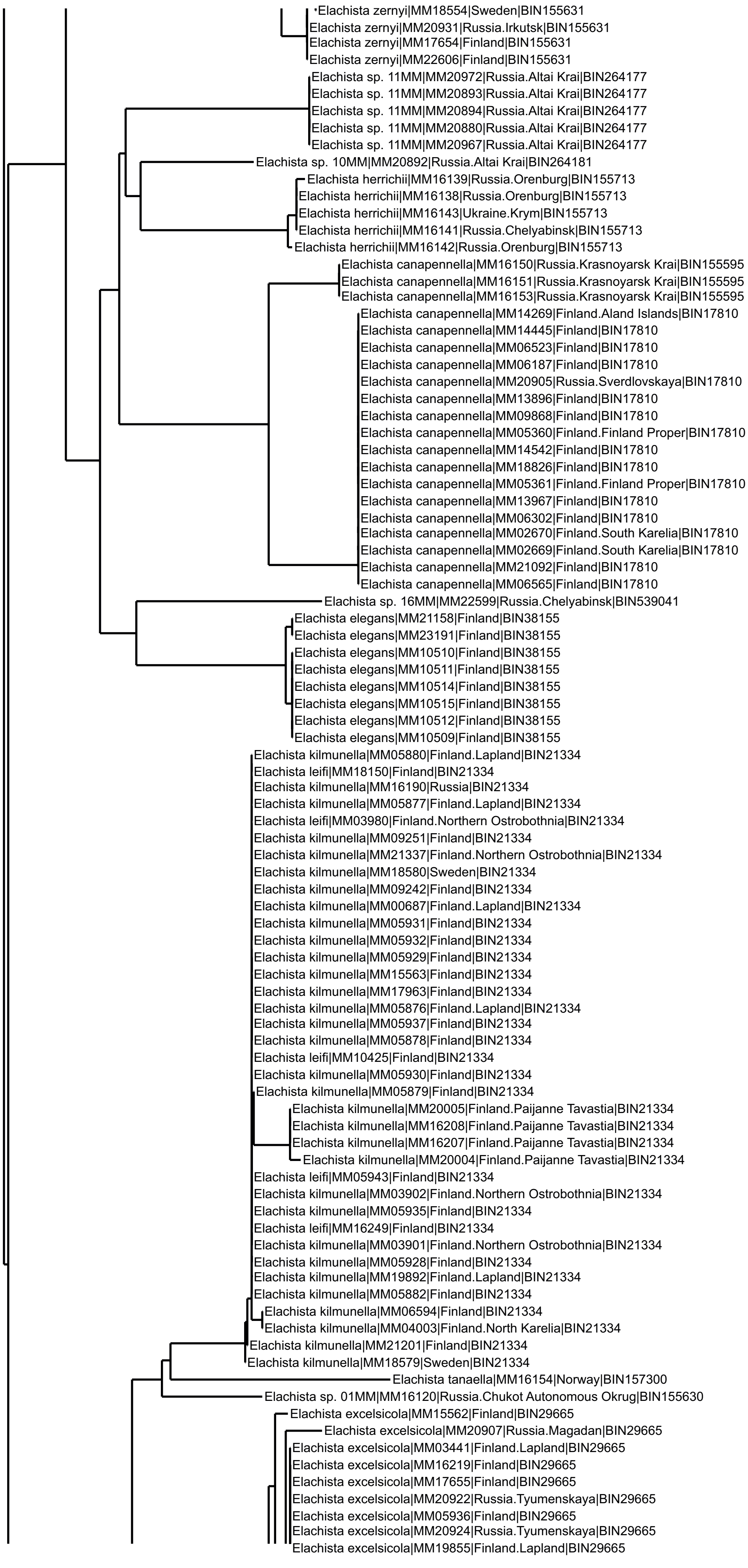
Elachista zernyi|MM18554|Sweden|BIN155631
Elachista zernyi|MM20931|Russia.Irkutsk|BIN155631
Elachista zernyi|MM17654|Finland|BIN155631
Elachista zernyi|MM22606|Finland|BIN155631
Elachista sp. 11MM|MM20972|Russia.Altai Krai|BIN264177
Elachista sp. 11MM|MM20893|Russia.Altai Krai|BIN264177
Elachista sp. 11MM|MM20894|Russia.Altai Krai|BIN264177
Elachista sp. 11MM|MM20880|Russia.Altai Krai|BIN264177
Elachista sp. 11MM|MM20967|Russia.Altai Krai|BIN264177
Elachista sp. 10MM|MM20892|Russia.Altai Krai|BIN264181
Elachista herrichii|MM16139|Russia.Orenburg|BIN155713
Elachista herrichii|MM16138|Russia.Orenburg|BIN155713
Elachista herrichii|MM16143|Ukraine.Krym|BIN155713
Elachista herrichii|MM16141|Russia.Chelyabinsk|BIN155713
Elachista herrichii|MM16142|Russia.Orenburg|BIN155713
Elachista canapennella|MM16150|Russia.Krasnoyarsk Krai|BIN155595
Elachista canapennella|MM16151|Russia.Krasnoyarsk Krai|BIN155595
Elachista canapennella|MM16153|Russia.Krasnoyarsk Krai|BIN155595
Elachista canapennella|MM14269|Finland.Aland Islands|BIN17810
Elachista canapennella|MM14445|Finland|BIN17810
Elachista canapennella|MM06523|Finland|BIN17810
Elachista canapennella|MM06187|Finland|BIN17810
Elachista canapennella|MM20905|Russia.Sverdlovskaya|BIN17810
Elachista canapennella|MM13896|Finland|BIN17810
Elachista canapennella|MM09868|Finland|BIN17810
Elachista canapennella|MM05360|Finland.Finland Proper|BIN17810
Elachista canapennella|MM14542|Finland|BIN17810
Elachista canapennella|MM18826|Finland|BIN17810
Elachista canapennella|MM05361|Finland.Finland Proper|BIN17810
Elachista canapennella|MM13967|Finland|BIN17810
Elachista canapennella|MM06302|Finland|BIN17810
Elachista canapennella|MM02670|Finland.South Karelia|BIN17810
Elachista canapennella|MM02669|Finland.South Karelia|BIN17810
Elachista canapennella|MM21092|Finland|BIN17810
Elachista canapennella|MM06565|Finland|BIN17810
Elachista sp. 16MM|MM22599|Russia.Chelyabinsk|BIN539041
Elachista elegans|MM21158|Finland|BIN38155
Elachista elegans|MM23191|Finland|BIN38155
Elachista elegans|MM10510|Finland|BIN38155
Elachista elegans|MM10511|Finland|BIN38155
Elachista elegans|MM10514|Finland|BIN38155
Elachista elegans|MM10515|Finland|BIN38155
Elachista elegans|MM10512|Finland|BIN38155
Elachista elegans|MM10509|Finland|BIN38155
Elachista kilmunella|MM05880|Finland.Lapland|BIN21334
Elachista leifi|MM18150|Finland|BIN21334
Elachista kilmunella|MM16190|Russia|BIN21334
Elachista kilmunella|MM05877|Finland.Lapland|BIN21334
Elachista leifi|MM03980|Finland.Northern Ostrobothnia|BIN21334
Elachista kilmunella|MM09251|Finland|BIN21334
Elachista kilmunella|MM21337|Finland.Northern Ostrobothnia|BIN21334
Elachista kilmunella|MM18580|Sweden|BIN21334
Elachista kilmunella|MM09242|Finland|BIN21334
Elachista kilmunella|MM00687|Finland.Lapland|BIN21334
Elachista kilmunella|MM05931|Finland|BIN21334
Elachista kilmunella|MM05932|Finland|BIN21334
Elachista kilmunella|MM05929|Finland|BIN21334
Elachista kilmunella|MM15563|Finland|BIN21334
Elachista kilmunella|MM17963|Finland|BIN21334
Elachista kilmunella|MM05876|Finland.Lapland|BIN21334
Elachista kilmunella|MM05937|Finland|BIN21334
Elachista kilmunella|MM05878|Finland|BIN21334
Elachista leifi|MM10425|Finland|BIN21334
Elachista kilmunella|MM05930|Finland|BIN21334
Elachista kilmunella|MM05879|Finland|BIN21334
Elachista kilmunella|MM20005|Finland.Paijanne Tavastia|BIN21334
Elachista kilmunella|MM16208|Finland.Paijanne Tavastia|BIN21334
Elachista kilmunella|MM16207|Finland.Paijanne Tavastia|BIN21334
Elachista kilmunella|MM20004|Finland.Paijanne Tavastia|BIN21334
Elachista leifi|MM05943|Finland|BIN21334
Elachista kilmunella|MM03902|Finland.Northern Ostrobothnia|BIN21334
Elachista kilmunella|MM05935|Finland|BIN21334
Elachista leifi|MM16249|Finland|BIN21334
Elachista kilmunella|MM03901|Finland.Northern Ostrobothnia|BIN21334
Elachista kilmunella|MM05928|Finland|BIN21334
Elachista kilmunella|MM19892|Finland.Lapland|BIN21334
Elachista kilmunella|MM05882|Finland|BIN21334
Elachista kilmunella|MM06594|Finland|BIN21334
Elachista kilmunella|MM04003|Finland.North Karelia|BIN21334
Elachista kilmunella|MM21201|Finland|BIN21334
Elachista kilmunella|MM18579|Sweden|BIN21334
Elachista tanaella|MM16154|Norway|BIN157300
Elachista sp. 01MM|MM16120|Russia.Chukot Autonomous Okrug|BIN155630
Elachista excelsicola|MM15562|Finland|BIN29665
Elachista excelsicola|MM20907|Russia.Magadan|BIN29665
Elachista excelsicola|MM03441|Finland.Lapland|BIN29665
Elachista excelsicola|MM16219|Finland|BIN29665
Elachista excelsicola|MM17655|Finland|BIN29665
Elachista excelsicola|MM20922|Russia.Tyumenskaya|BIN29665
Elachista excelsicola|MM05936|Finland|BIN29665
Elachista excelsicola|MM20924|Russia.Tyumenskaya|BIN29665
Elachista excelsicola|MM19855|Finland.Lapland|BIN29665

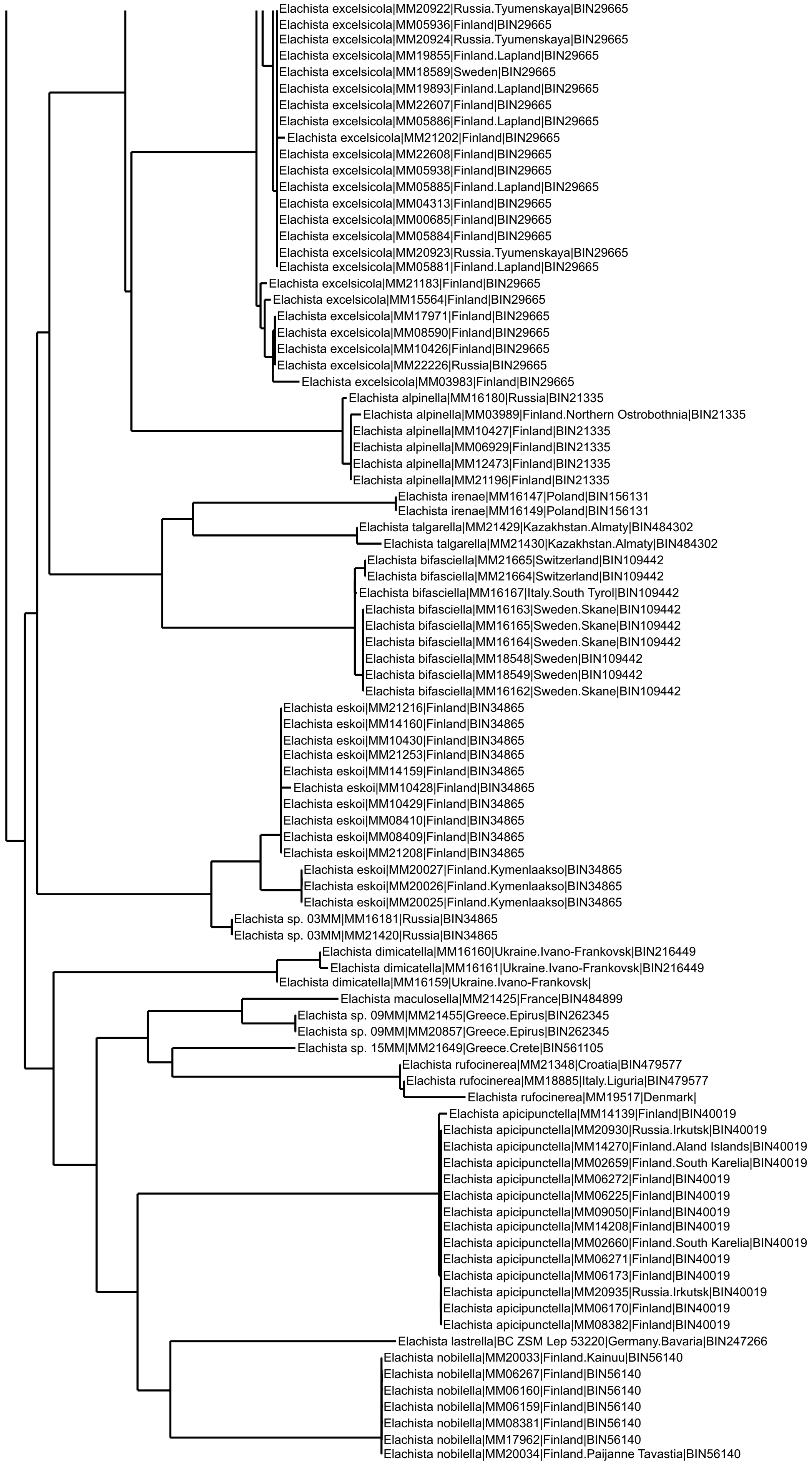

Figure S2. A detailed Neighbor-Joining tree, generated under the K2P nucleotide substitution model, of the study taxa with BIN numbers indicated for each specimen. The tree includes the outgroup species (*E. abiskoella*) but is not rooted to it.
